# Targeting the Microbiota Reverses C‐Section‐Induced Effects on Intestinal Permeability, Microbiota Composition, and Amygdala Gene Expression in the Mouse

**DOI:** 10.1111/nmo.70107

**Published:** 2025-06-26

**Authors:** Ana Paula Ventura Silva, Gerard M. Moloney, Ana Marta Sequeira, Marta Liber, Thomaz Bastiaanssen, Kieran Rea, Patrick Fitzgerald, Anna Golubeva, Maria Rodriguez‐Aburto, Ingrid B. Renes, Jan Knol, Timothy Dinan, John F. Cryan

**Affiliations:** ^1^ Department of Anatomy and Neuroscience University College Cork Cork Ireland; ^2^ APC Microbiome Ireland University College Cork Cork Ireland; ^3^ Danone Nutricia Research Utrecht the Netherlands; ^4^ Department of Psychiatry and Neurobehavioural Science University College Cork Cork Ireland

**Keywords:** amygdala, caesarean‐section, diet, microbiome, neuroplasticity, permeability

## Abstract

**Background:**

The microbiome significantly influences the development of the gastrointestinal and immune systems. The delivery method, whether Caesarean section (CS) or vaginal birth (VB), plays a crucial role in shaping microbiota composition, with CS babies exhibiting differences. Early‐life nutritional interventions using probiotics or prebiotics may help restore this imbalance in CS infants. Our study aimed to assess gut permeability in CS mice compared to VB mice and explore whether prebiotics or probiotics could mitigate any deficiencies.

**Methods:**

Using a mouse model (NIH Swiss) for CS delivery, we measured plasma levels of a 4 kDa macromolecule (FITC) at PND7, 14, 23, and 35. We evaluated ileal gene expression of tight junction proteins, profiled intestinal microbiome composition, and examined the expression of genes involved in neurotransmitter physiology in the amygdala. Additionally, we studied the impact of administering *Bifidobacterium breve* in drinking water and dietary administration of GOS/FOS on these outcomes.

**Key Results:**

At PND7, CS‐born mice exhibited increased ileal permeability, along with reduced expression of Tjp1, Occludin, Claudin 3, and Epcam compared to VB mice. Administration of *B. breve* or GOS/FOS alleviated changes in Epcam expression. During the pre‐weaning period, beta diversity differed between VB and CS. Post‐weaning, β‐diversity increased following probiotic and prebiotic intervention. Additionally, CS mice showed changes in neurotransmitter gene expression in the amygdala, which were also mitigated by *B. breve* or GOS/FOS.

**Discussion:**

Our findings indicate that targeted microbiota‐associated interventions can reverse deficits in intestinal permeability induced in CS mice.

## Graphical abstract

The study investigated the impact of the delivery method on the microbiome in mice, revealing increased intestinal permeability and altered gene expression in Caesarean section (CS)‐born mice compared to vaginal birth (VB) mice. Administration of *Bifidobacterium breve* or GOS/FOS partially mitigated these effects, suggesting targeted microbiota interventions can reverse deficits in intestinal permeability induced by CS.

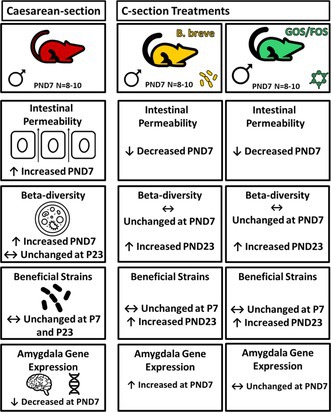

**Summary**

- Mode of delivery can significantly shape the composition of the gut microbiota. This study investigated how Caesarean section (CS) birth affects gut permeability, microbiota, and gene expression in the amygdala of mice and whether early‐life interventions with probiotics or prebiotics can reverse these effects.
- Using NIH Swiss mice, we found that CS‐born pups had higher gut permeability and reduced expression of tight junction genes at PND7 compared to vaginally born (VB) mice. Probiotic (*Bifidobacterium breve*) and prebiotic (GOS/FOS) treatments improved gene expression and modulated the microbiota, especially increasing diversity after weaning.
- CS birth also altered neurotransmitter‐related gene expression in the amygdala, changes that were mitigated by both interventions.
- These findings suggest that early probiotic or prebiotic supplementation may help restore gut and brain development disruptions linked to CS delivery in mice.

## 1 Introduction

The intestinal barrier is an essential structure at the interface between the host and the external environment. This barrier protects the underlying mucosa, maintaining systemic immune tolerance, protecting the host from continuous attacks from pathogens and antigens, while also facilitating the uptake of essential nutritional molecules that are key to host survival [1, 2]. To accomplish such diverse functions, the gut possesses many structural and immune mediated mechanisms that facilitate host protection. Firstly, the physical propulsion of the GI tract protects underlying structures [3]. Secondly, commensal microbes prevent the permanent colonization of pathogenic bacteria [4, 5]. Thirdly, immune cells in the mucosa and *lamina propria*, such as macrophages and dendritic cells, mount potent immune responses against pathogens via the production of cytokines and immunoglobulins [6, 7]. Moreover, a protective mucus layer allows the entrapment of pathogenic bacteria and prevents direct contact with the epithelial cell layer [8]. Finally, the epithelium, a single layer of epithelial cells sealed by tight junction proteins, desmosomes, and adherens junctions, provides a robust physical barrier preventing access to the underlying mucosa [9].

The mammalian microbiome consists of over 100 trillion microorganisms and has co‐evolved with its host over decades, forming a mutualistic relationship now accepted to play a key role in the development of the immune system [10], host metabolism [11], and the maintenance of the structural integrity of the intestinal barrier [12]. It is clear that the microbiome in our gut influences key brain structures and behavior [13, 14]. Changes in the composition of the microbiome often occur in early life; for example, feeding—whether with breast milk or formula—can influence the development and structure of the gut microbiota along with neural development [15, 16]. Antibiotics also have a profound effect on the microbiome, including reduced species diversity and impeded growth of *Bifidobacterium* [17].

Colonization of neonates with environmental microorganisms and microorganisms from their mothers begins at birth and is critical for development. Caesarean section (CS) is an essential surgical procedure when vaginal delivery endangers the mother or the infant. Moreover, delivery by CS along with infant feeding and antibiotic use is a primary contributor to disruption of the infant microbiome [18]. This manifests as decreased colonization of *Bacteroides*, *Lactobacillus*, and *Bifidobacterium* and an increase in the presence of *Streptococcus, Staphylococcus, and Propionibacterium*—strains normally associated with the skin microbiome [18, 19, 20, 21, 22]. Disturbing the natural transmission of bacterial species from mother to infant is known to have detrimental consequences later in life. CS delivery is now associated with an increased relative risk of obesity [23], asthma [24, 25], Type 1 diabetes [26], and coeliac disease [27].

Like the microbiome, development of the intestinal barrier is a dynamic process and is incomplete at birth. In fact, increased intestinal permeability is considered a normal finding in the neonatal intestine [28, 29]. Increased permeability may be beneficial to the neonate, facilitating the uptake of larger nutritional peptides [30]. However, increased permeability may also result in increased uptake of antigen and pathogenic microbes causing infection and increased visceral hypersensitivity. A defective intestinal barrier and an altered microbiome composition are also associated with intestinal disorders such as irritable bowel syndrome (IBS) [31], and inflammatory bowel disease (IBD) [32]. Nutritional interventions using supplementation with probiotics, prebiotics, synbiotics, or postbiotics from early life may restore deficits in the microbiota of CS offspring and prevent the detrimental effects associated with this mode of delivery [33].

Using a mouse model of CS, we have previously shown social and cognitive deficits in early life that persist into adulthood compared to vaginally born mice [34]. We have also demonstrated that many of these alterations can be reversed with dietary interventions using prebiotics (FOS/GOS) [35] or a specific Bifidobacterium [34]. Thus, in this study, we sought to profile intestinal barrier function at various stages of early life following CS and assess whether any potential deficits were reversable by pre‐ or probiotic intervention. In addition, we assessed the effects of CS birth on the expression of genes relevant to neuroplasticity in the amygdala, while also considering the role of the intestinal microbiome.

## 2 Methods

### 2.1 Animals and Housing

Male and female NIH Swiss mice breeders (Hsd: NIHS) were purchased at 9 weeks old from Envigo UK. An outbred strain was used because of better maternal behavior compared to inbred strains [36]. Animals were allowed to acclimatize to the animal holding room for up to 2 weeks before breeding started. Pregnant female mice were housed individually throughout pregnancy and had *ad libitum* access to regular rodent diet (Teklad Global 18% rodent diet, 2018s) and tap water. Mice were housed in conventional cages (M2 polypropylene, sloping front cages. Overall size 33 x 15 x 13 cm. Internal size 330 cm x 13 cm). Bedding consisted of eco‐pure aspen premium bedding with sizzlenest, nesting material (Datesand). Each cage was also supplied with a cardboard tube and chew stick. See Table S3 for ARRIVE guidelines.

Pregnant females (primiparous) were time mated in the presence of a vaginal plug indicating day 0.5 of gestation. Three days before the breeding was due to begin, dirty bedding from male mice was placed in the females cage to promote the Whitten Effect. Males and females were then paired. Vaginal plugs were checked for daily. Once a plug was detected, the female was removed and single housed. If a plug was not detected, males were removed and paired with a different female. Females were presumed pregnant for 3 weeks, at which stage they are reused for breeding. Female cages were not changed 4 days before their due date and for up to a week after they gave birth. Male offspring were weaned at postnatal day (PND) 23 and subsequently housed in groups of 3 or 4 per cage. Full nests were maintained until weaning; at weaning, female mice were euthanized, and male mice were retained. Animals were kept under controlled temperature and humidity (20 ± 1 ± °C, 55.5%) and on a 12‐h (h) light, 12 h dark cycle (lights on from 07h30‐19h30). All experiments were conducted during the light phase, and all procedures complied with the European Directive 2010/63/EU, compliant with the requirements of Irish law (S.I No 543 of 2012) and approved by the Animal Experimentation Ethics Committee of University College Cork and the Health Products Regulatory Authority (HPRA). Approval was obtained before the commencement of any of the experiments.

### 2.2 C‐Section

At day 19.5 of gestation (mean 20 days ±1 day), pregnant females were culled by cervical dislocation and C‐section surgery was performed. Briefly, after cleaning the abdominal area with 70% ethanol, an incision was made in the abdominal cavity to expose the uterus. Individual pups were gently removed from the uterus, the umbilical cord was cut approximately 3 mm distal to the umbilical attachment and pups were placed on sterile gauze on top of a heating pad. Sterile cotton buds were used to gently massage the pups until spontaneous breathing was observed. At the end of the procedure, all the pups were given to a foster mother that had a naturally delivered litter within the previous 24 h.

### 2.3 Study Design and Treatments

This study was designed with four specific timepoints: P7, P14, P23 and P35, all samples from these timepoints were analyzed at the very end of the study. These timepoints were chosen to encompass, early life and post‐weaning periods. Different animals were used at each timepoint, and each individual animal was considered an experimental unit. To address litter effects, we randomly allocated animals from 4–6 litters to groups as outlined in Tables S3 and S4.

Mice were divided into four experimental groups: one natural born (NB) group receiving water and control AIN diet (Ssniff diets, Table S2A **)** and three Caesarean delivery (CS) groups receiving one of the following treatments: 1. CS Control; 2. *Bifidobacterium Breve M16V* (probiotic) a commercially available probiotic supplied by Danone Nutricia Research (Utrecht, the Netherlands). Group 3, a prebiotic mixture of short‐chain galacto‐oligosaccharides (GOS) and long‐chain fructo‐oligosaccharides (FOS) in a 9 to 1 ratio supplied by Danone Nutricia Research (Utrecht, the Netherlands), (GOS/FOS, prebiotic), (Table S2B). Dietary intervention was given *ad‐libitum* to (foster) dams from birth throughout the lactation period. For the postnatal day (PND) 35 timepoint, male offspring were weaned at PND 21 and moved to the same dietary intervention as their mothers until the end of the experiment. *B. breve* treatment was administered in the drinking water, and GOS/FOS was included in the custom rodent diet, which was refreshed daily. *B. breve* solution was prepared fresh every day at a concentration of 1x10 [9]/mL, with the probiotic being dissolved in sterile H_2_O (Medidrop).

### 2.4 In Vivo Gastrointestinal Permeability (Fluorescein Isothiocyanate (FITC)‐Dextran)

For PND7 and PND14, mice were separated from their mother and placed on a heating pad for 1 h to avoid hypothermia. The heating pad was under the cage placed in a separate room to the dam, away from sights and smells of the dams. This room was quiet and had standard lighting. After 1 h, FITC‐dextran (600 mg/kg, diluted in sterile PBS) was given by oral gavage. For PND 7 and 14 mice were culled 2 h after gavage and FITC was measured in the plasma. For PND23 and PND35 mice were fasted overnight before being given FITC‐dextran by oral gavage (600 mg/kg). 2 h after gavage, blood was collected following decapitation and FITC was measured in the plasma at 485‐nm excitation and 535‐nm emission wavelengths in a 384‐well plate.

### 2.5 Fecal Collections

Colon content was collected for microbiome analysis when the animals were culled.

### 2.6 16S rRNA Gene Sequence‐Based Microbiota Analysis

Total DNA extraction from fecal matter from the colon was performed using the QIAmp Fast DNA Stool mini‐Kit (QIAGEN, Manchester, UK) coupled with an initial bead‐beating step. Extracted DNA was kept frozen at −20°C until further analysis. The V3‐V4 hypervariable region of the 16S rRNA gene was amplified with primers Bact‐0341F (5′‐CCTACGGGNGGCWGCAG‐3′) and Bact‐0785R (5′‐GACTACHVGGGTATCTAATCC‐3′) [37]. PCR products were quantified, normalized and pooled in an equimolar fashion using Invitrogen kit Quant‐It‐Picogreen (ThermoFisher Scientific, Oregon, USA) and prepared for sequencing on an Illumina MiSeq instrument as described previously (Caporaso, Lauber et al. 2012) using NexteraXT library preparation kit (Illumina, San Diego, USA) and MiSeq Reagent Kit v3 (Illumina, San Diego, USA; 300 PE reads).

### 2.7 Bioinformatics Analysis

Three hundred base pair paired‐end reads were pre‐filtered on the basis of a quality score threshold of > 28, trimmed and filtered for quality and chimaeras using the DADA2 library in R (version 4.1.1). Only samples with > 10.000 reads after QC were used in analysis. Taxonomy was assigned with DADA2 against the SILVA SSURef database release v132. Parameters as recommended in the DADA2 manual were adhered to unless mentioned otherwise. Amplicon sequence variants (ASVs) were aggregated at genus level. Raw microbiome files are deposited in the ENA under accession number PRJEB81708. Raw Data is available in Supplementary Data S1, S2 and S3.

### 2.8 Quantitative Real Time Polymerase Chain Reaction (qRT‐PCR)

Gut (colon and ileum) and brain (amygdala) tissue were rapidly hand‐dissected and then stored at −80°C until further tissue processing. RNA extraction was performed using the MirVANA Total RNA Isolation Kit (Ambion) according to the manufacturer's instructions. For cDNA synthesis, the Applied Biosystems High‐Capacity cDNA Reverse Transcription Kit (Life Technologies) was used per the instructions provided by the supplier. qRT‐PCR was performed with 7300 Real‐time PCR systems (Applied biosystems), using TaqMan Universal Mastermix II, no UNG (Applied Biosystems) and PrimeTime std. qPCR Assay primers (Integrated DNA Technologies). All samples were measured in triplicate. β‐actin (Actb) was used as a stable reference gene and to normalize the expression of the different genes [38].

### 2.9 Statistics

In this study, a single animal was considered the experimental unit. Data distribution was checked by the Kolmogorov–Smirnov test, and variances were compared using Levene's test. For parametric data, a Student's *t*‐test and a one‐way ANOVA followed by Bonferroni post hoc were applied accordingly. For nonparametric data, a Kruskal–Wallis test followed by the U‐Mann–Whitney test was used. The experimental unit for all analyses was each single animal. All statistical analyses were carried out using IBM SPSS Statistics 25.0 for Windows software package. Extreme outliers and technical outliers were excluded when values were 2× standard deviation from the mean.

For the microbiome analysis, we excluded species that were only detected as non‐zero in 5% or fewer of total samples from our count table as ratios are invariant to subsetting and this study employs compositional data analysis techniques [39]. The ALDEx2 library was used to compute the centred log‐ratio transformed values of the remaining taxa as is appropriate for handling compositional data (Fernandes, Reid et al. 2014). Custom scripts and the Tjazi library can be found online at https://github.com/thomazbastiaanssen/Tjazi [40]. For principal component analysis (PCA), a pairwise implementation of the adonis() PERMANOVA function in the vegan library followed by the Bonferroni‐Holm correction was used to test for difference in β‐diversity in terms of Aitchison distance (Aitchison, Barceló‐Vidal et al. 2000).

To statistically assess differences in microbial genera, we used the default (e.g., unpaired, non‐parametric) settings for ALDEx2 to estimate *p*‐values from permutations. This could be interpreted as a bootstrapped Mann–Whitney test [41]. We used Benjamini & Hochberg's procedure to control the false discovery rate (FDR). In all cases, a *q*‐value < 0.1 was considered significant. α‐diversity was calculated using the iNEXT library, and differences between treatment groups were assessed using a linear model [42].

## 3 Results

### 3.1 Delivery by C‐Section Increases Intestinal Permeability at Postnatal Day 7 and is Reversible With *B. breve* or GOS/FOS Treatment

**FIGURE 1:**
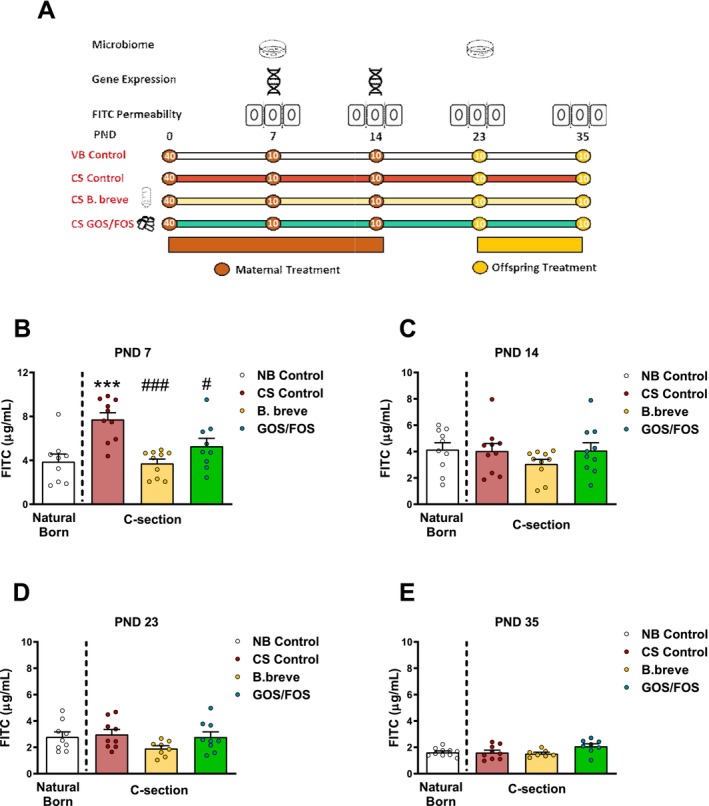
Delivery by C‐Section increases intestinal permeability in early life and is reversible with *B. breve* or GOS/FOS treatment. (A) Experimental timeline measurement of plasma FITC μg/mL at PND7 (B) PND14 (C) PND23 (D) and PND35 (E) in VB and CS mice supplemented with *B. breve* (yellow) or GOS/FOS (green). Data is presented as bar graphs with individual data points and error bars represents the standard error of the mean (S.E.M) *** = *p* < 0.001 versus VB control, # = *p* < 0.05 versus VB control, ### = *p* < 0.001 versus CS control. CS, C‐section birth; PND, postnatal day; VB, vaginal birth.

To investigate if mode‐of‐delivery at birth could influence early life intestinal permeability, we measured the presence of orally administered dextran conjugated with FITC in the plasma of mice at PND7, PND14, PND23, and PND35 (Figure 1A). Comparing the level of FITC in the plasma of mice born *per vaginum* with mice born by CS, we observed a significant increase in the concentration of FITC in mice born by CS at postnatal day 7 (Figure 1B, *p* < 0.001). The concentration of FITC in the plasma of mice did not differ on the basis of mode‐of‐delivery at days 14, 23 and 35 days following birth (Figure 1B). To assess if the increased intestinal permeability in CS mice at day 7 could be reversed with a microbiome targeted intervention, independent groups of mice were administered either a probiotic ( *B. breve* ) or a prebiotic (GOS/FOS) from birth maternally (up until PND23) or orally from PND23 to PND35, post‐weaning (Figure 1A). Mice who were born by CS and received *B. breve* (*p* < 0.001) or GOS/FOS (*p* < 0.05) had significantly reduced permeability at day 7 compared to mice born by CS receiving no dietary intervention. No improvements in intestinal permeability were noted in mice receiving *B. breve* or GOS/FOS at days 14 (Figure 1D), 23 (Figure 1E) or 35 (Figure 1F).

### 3.2 Delivery by C‐Section Reduces the Expression of Tight Junction Protein Related Genes Specifically at PND7 in the Ileum

**FIGURE 2:**
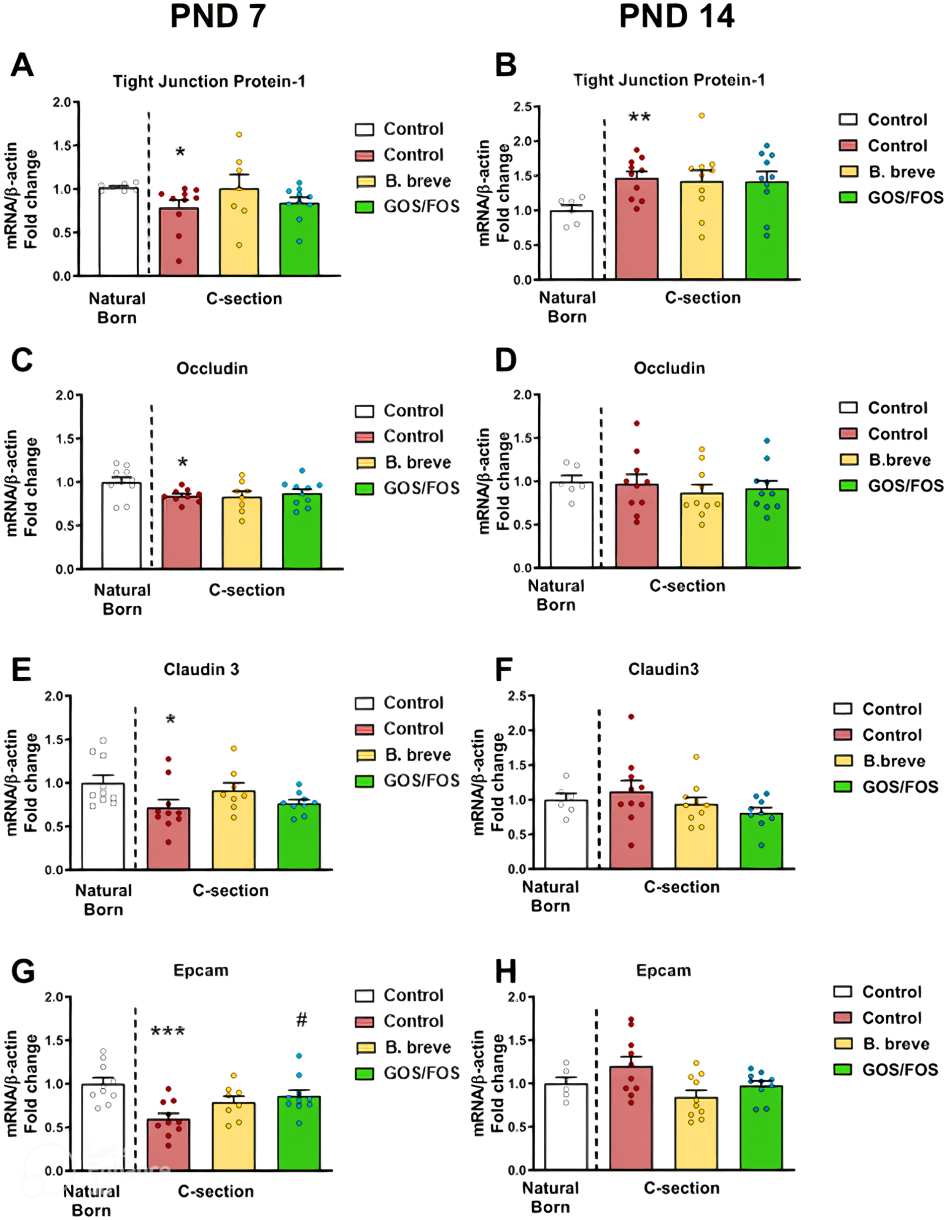
Delivery by C‐Section reduces the expression of tight junction protein related genes specifically at PND7 in the ileum. Relative expression of Tjp1 (A, B), Occludin (C, D), Claudin 3 (E, F), and Epcam (G, H) in VB (white), CS (red), CS supplemented with *B. breve* (yellow) and CS supplemented with GOS/FOS (green) at PND 7 (A, C, E, G) and PND14 (B, D, F, H). *N* = 8–10 per group. *** = *p* < 0.001 versus VB control, * = *p* < 0.05 versus VB control, ** = *p* < 0.01 versus VB control. # *p* < 0.05 versus CS control, CS, C‐section birth; VB, vaginal birth. PND 7 *N* = 10, 10, 10, and 8 per group, PND 14 *N* = 6, 10, 10, and 10 per group. Data are presented as bar graphs with individual data points and error bars representing the standard error of the mean (S.E.M).

Having shown that delivery by CS results in increased intestinal permeability compared with mice born vaginally at PND7, we speculated that this may be due to structural alterations in the epithelial barrier. In the ileum, we measured expression of genes associated with the structural integrity of the epithelial barrier. TJP1 (Tight Junction Protein 1), Occludin, and Claudin3 are transmembrane proteins with key structural roles in the maintenance of tight junctions between epithelial cells (Shen, Weber et al. 2011). At PND7, there was a significant decrease in the expression of TJP1, Occludin, and Claudin3 in mice born by CS (Figure 2A,C,E, *p* < 0.05) compared with VB mice. Subsequently, 14 days after birth, TJP1 was found to be significantly increased in the intestine of mice born by CS (Figure 2B, *p* < 0.01), whereas CS did not affect the expression of Occludin or Claudin3 at this time (Figure 2D,F). Epithelial cell adhesion molecule (Epcam) is a transmembrane protein expressed almost exclusively in epithelial cells, where it plays a critical role in cell adhesion (Lei, Maeda et al. 2012). At PND7, expression of Epcam was significantly decreased in mice who had been born by CS (Figure 1G, *p* < 0.001) compared with VB mice. However, at day 14, expression of Epcam was similar between CS and vaginal delivery (Figure 2H). *B. breve* and GOS/FOS did not significantly alter the expression of TJP1, Occludin or Claudin3 compared to mice born by CS. Expression of Epcam in CS mice receiving GOS/FOS was significantly higher compared to mice born by CS without any treatment (Figure 2G). Collectively, these data suggest that deficits in tight‐junction gene expression observed in CS mice at PND7 are not on the whole amenable to interventions that target the microbiome.

### 3.3 C‐Section Alters Gut Microbiota at PND7. At PND23, Treatment With *B. breve* or GOS/FOS has a Significant Impact on Beta Diversity

**FIGURE 3:**
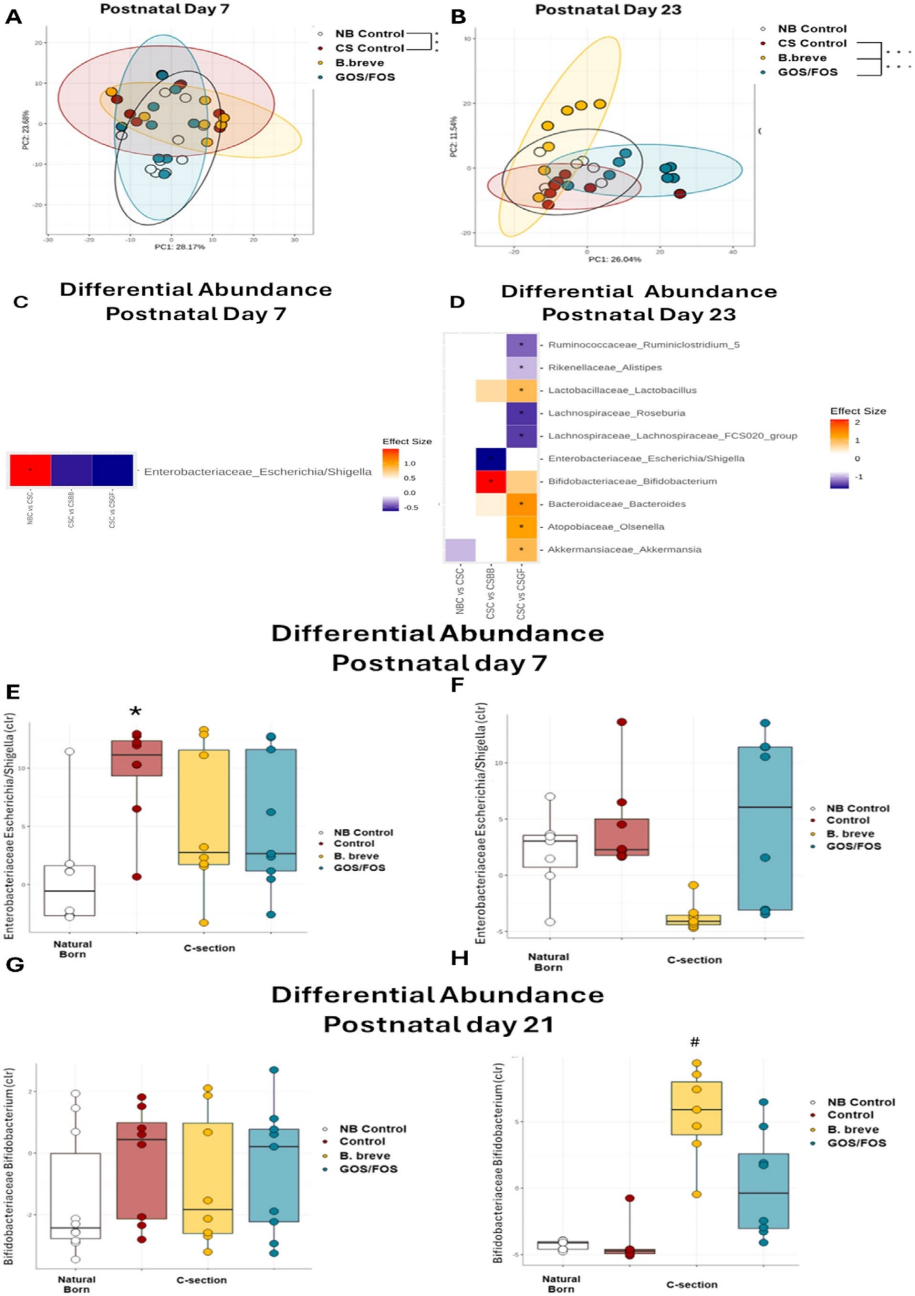
C‐Section alters gut microbiota at PND7. At PND23, treatment with *B. breve* or GOS/FOS has a significant impact on beta diversity. (A, B) Principal component analysis (PCA) showing the effects of mode of delivery and supplementation with prebiotic or synbiotic on microbiome composition at PND 7 (A) and PND23 (B). (C, D) Heat map showing genera differentially altered by mode of delivery. (C, D) Differentially abundant taxa (by glm Beta coefficient). Color depicts effect size, with blue (negative) indicating higher abundances pre‐treatment and red (positive) indicating higher abundances post‐treatment. *q < 0.1. Day 7, *N* = 10, 8, 8, and 9 per group, Day 23 *N* = 7, 8, 7, and 8 per group. (E, F) Representative boxplots of Enterobacteriaceae_Escherichia/Shigella centred log‐ratio (clr) transformed abundance at PND7 (E) and PND23 (F) and Bifidobacteriaceae_Bifidobacterium clr transformed abundance at PND7 (G) and PND23 (H) taken from the feces of VB (white), CS (red), CS supplemented with *B. breve* (yellow) and CS supplemented with GOS/FOS (green).

The reduced barrier function or increased permeability is inherently linked with the composition of the gut microbiome as it is at this interface that most host–microbe interactions occur. Having observed a temporal effect of birth by CS on barrier integrity at PND7 and the capacity of two microbiota‐based interventions to reverse this deficit in permeability, we analyzed the intestinal microbiome at PND7 and PND23 (post‐weaning) in mice born vaginally, by CS and in mice born by CS receiving *B. breve* or GOS/FOS (Figure 3). Alpha‐diversity was not significantly different between CS and VB at PND7 and PND23 using the Chao1, Shannon and Simpson indices (Table S1), whereas β‐diversity was assessed in terms of Aitchison distance and visualized via PCA. We found modest differences at PND7 in microbiome composition between mice born vaginally and mice born by CS (Figure 3A, *p* < 0.008, *R* ^2^ 0.017, *F* value 3.47), whereas treatment with GOS/FOS (*p* < 0.15, *R* ^2^ 0.09, *F* value 1.49) or treatment with *B. breve* (*p* < 0.14, *R* ^2^ 0.1, *F* value 1.68) did not show significant changes in composition compared to CS mice receiving no intervention.

At PND23, no changes in α‐diversity were noted using the Chao1, Shannon, and Simpson indices (Table S1). Assessing β‐diversity at PND23, there was no significant change in the composition of the microbiome between VB and CS mice (Figure 3B, *p* < 0.08, *R* ^2^ 0.11, *F* value 1.54). Treatment with *B. breve* (*p* < 0.002, *R* ^2^ 0.17, *F* value 2.71) and GOS/FOS (*p* < 0.003, *R* ^2^ 0.21, *F* value 3.64) significantly altered the composition of the microbiome compared to CS mice receiving no intervention (Figure 3B). When we assessed individual differential abundance at the genus level, at PND7, Enterobacteriaceae_Escherichia/Shigella was significantly decreased in VB mice compared with CS mice (*p* < 0.001, 95% CI (lwr − 9.64, upper 1.41, *F* value 4.98), (Figure 3C,E and Table S5), whereas treatment with *B. breve* or GOS/FOS did not affect abundance of this strain (Figure 3C,E). At PND23, CS did not result in significant changes in differential abundance of Enterobacteriaceae_Escherichia/Shigella, but, like the overall microbiome composition, treatment with *B. breve* or GOS/FOS increased the abundance of specific strains such as Bifidobacteriaceae_Bifidobacterium, Bacteroidaceae_Bacteroides and Akkermansiaceae_Akkermansia amongst others (Figure 3D,F and Table S6).

Intriguingly, different treatments had differential effects on specific strains. At PND23, comparing effect sizes, mice born by CS who received *B. breve* showed a trend towards a decrease in Enterobacteriaceae_Escherichia‐Shigella and a significant increase in Bifidobacteriaceae_Bifidobacterium abundance compared to CS mice who received no intervention (Figure 3D and Table S6). Interestingly, at PND23, the genera changed in mice born by CS that received GOS/FOS were different to those receiving *B. breve* , with Ruminococcaceae_Ruminiclostridium_5, Rikenellaceae_Alistipes, Lachnospiraceae_Roseburia and Lachnospiraceae_Lachnospiraceae_FCS020_group decreased and Lactobacillaceae_Lactobacillus, Bifidobacteriaceae_Bifidobacterium, Bacteroidaceae_Bacteroides, Atopobiaceae_Olsenella and Akkermansiaceae_Akkermansia increased in treated mice compared to untreated mice (Table S6).

### 3.4 Expression of Genes That Code for Neurotransmitters in the Amygdala Are Altered in Mice Born by C‐Section

**FIGURE 4:**
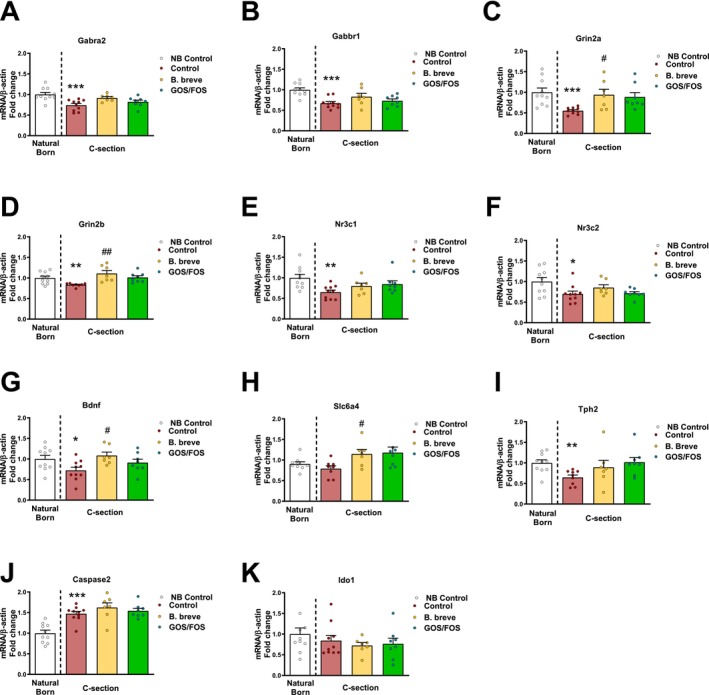
Expression of neurotransmitters in the Amygdala is altered in mice born by C‐section. Relative expression of Gabra2 (A) Gabbr1, (B) Grin2a, (C) Grin2a, (D) Grin2b, (E) Nr3c1, (F) Nr3c2, (G) Bdnf, (H) Slc6a4, (I) Tph2, (J) Caspase2, and (K) Ido1 was measured in the amygdala of VB (white), CS (red), CS supplemented with *B. breve* (yellow) and CS supplemented with GOS/FOS (green) at PND 7. *N* = 10, 10, 7, and 9 per group. *** = *p* < 0.001 versus VB control, # = *p* < 0.05 versus VB control, ** = *p* < 0.01 versus CS control, * = *p* < 0.05 versus CS control. # *p* < 0.05 versus CS control, ## *p* < 0.01 versus CS control. CS, C‐section birth; VB, vaginal birth.

Delivery by CS had a significant effect on the expression of genes related to several important neurotransmitters in the amygdala at PND7. In fact, expression of the GABA receptors, Gabra2 (Figure 4A, *p* < 0.001) and Gabbr1 (Figure 4B, *p* < 0.001), along with the glutamate transporters, Grin2a (Figure 4C, *p* < 0.001), and Grin2b (Figure 4D, *p* < 0.01), were significantly decreased in CS, as were the glucocorticoid receptor Nr3c1 (Figure 4E, *p* < 0.01) and the mineralocorticoid receptor Nr3c2 (Figure 4F, *p* < 0.05). Brain‐derived neurotrophic factor (Bdnf), a modulator of neurotransmission, was decreased in CS compared with VB mice (Figure 4G, *p* < 0.05), whereas the expression of the serotonin transporter (Sert or SLC6A4) was not significantly different between mice born vaginally or by CS (Figure 4H). Interestingly, the gene that encodes Tph2, an enzyme responsible for serotonin synthesis in the amygdala, was significantly decreased in CS mice compared to NB mice (Figure 4I, *p* < 0.01). Caspase‐2, a regulator of synaptic pruning, was significantly increased in CS mice compared to VB mice (Figure 4J, *p* < 0.001) and the expression of Ido1, a gene encoding an enzyme in the tryptophan metabolism pathway, was not changed on the basis of mode‐of‐delivery (Figure 4K).

Having shown that mode‐of‐delivery affects the expression of genes related to various neurotransmitters in the amygdala, we wanted to investigate if *B. breve*, or GOS/FOS, were able to reverse such changes. Treating mice born by CS with the probiotic *B. breve* increased the expression of the genes Grin2a, Grin2b, Bdnf, and Slc6a4, in the amygdala compared to untreated CS mice (Figure 4). Treatment with *B. breve* did not affect the expression of Gabra2, Gabbr1, Nr3c1, Nr3c2, Tph2, Caspase‐2 and Ido1. Importantly, these data suggest that gene expression in the amygdala is potentially influenced by mode‐of‐delivery and, of further interest, deficits in the expression of some of these genes related to key neurotransmitters, such as Grin2a, Grin2b, Bdnf and Sert, appear to be reversed by treatment with probiotic intervention ( *B. breve* ), but not a prebiotic (GOS/FOS). Further work should confirm these findings and investigate the precise mechanisms responsible. These data indicate that different interventions that alter the microbiome do so through different mechanisms, which in turn could lead to varying outcomes in the gut, but also in the brain. Further work is required to confirm this.

## 4 Discussion

We previously showed that CS birth disrupts maternal microbial transmission and immune priming in mice, but its impact on intestinal barrier function is unclear. Since barrier defects are linked to chronic gut and brain diseases, we examined early‐life permeability and found it increased in CS mice at PND7. CS mice also had altered tight junction gene expression, microbiome profiles, and amygdala neurotransmitter levels. To explore the microbiome's role, we tested a probiotic ( *B. breve* ) and prebiotic (GOS/FOS), both of which restored barrier function and normalized gene expression at PND7.

The influence of CS birth on immunity, intestinal morphology and the gut–brain axis is not completely understood, and much of the work in this field has focused on microbial composition and colonization at birth and in early life [34, 43]. A key site for host–microbe interactions is the intestinal barrier, which controls permeability, innate immune function, and mucous production [44]. Using two different techniques, we confirmed that CS delivery influences the structure and permeability of the gut barrier.

Tight junction proteins are important in maintaining the intestinal barrier by limiting paracellular permeability and are regulated by the microbiome [44]. We selected Tjp1, Occludin, and Claudin3 to interrogate barrier function in the ileum as they are key structures in the gut barrier and are responsive to changes in the microbiome [45, 46]. Future studies should also examine other tight‐junction proteins.

In mice born by CS, it is not clear what effects early life fluctuations in barrier permeability have on later life. In adulthood, expression of ileal Tjp1 is increased in CS mice [47], whereas we show that even as early as PND14, Tjp1 expression is increased in CS mice, highlighting the changeable nature of the intestinal barrier at this time. The period between birth and weaning is a critical developmental window when bacteria and bacterial products regulate the development and imprinting of the immune system, which persists into adulthood [48].

CS birth results in different patterns of colonization, which in turn can regulate the priming of gut immunity in rodents and humans [47, 49, 50]. Given that permeability is altered at different stages of development in CS mice, and the composition of the microbiome also differs from that of VB mice at similar times across development [34], it is tempting to speculate that intestinal permeability and the microbiome are interconnected in the results shown here [51]. We have previously shown that the caecal microbiome of mice is highly variable in early life (PND9) compared to adolescence (PND21) and adulthood (week 20) [34], and likewise, the infant microbiome is highly dynamic during the neonatal period, with mode‐of‐delivery being the most significant driver of variation during the neonatal period [22]. The microbiome is clearly linked to intestinal barrier function. Germ‐free mice have a thinner mucus layer, allowing for greater microbial access to the mucosa [52, 53]. Mice deficient in intestinal alkaline phosphatase (IAP), an antimicrobial protein situated at the intestinal villus brush‐border, have fewer bacteria and reduced microbial diversity [54]. Epithelial cell renewal is reduced in germ‐free and antibiotic‐treated mice, whereas microbial metabolites inhibit stem cells in the intestinal crypt [55].

We analyzed the microbial composition of feces in the ileocolonic region [47]. Similar to the caecal microbiome in early life (PND9), we see significant differences in the composition of the ileal microbiome in CS mice compared to VB mice [34] and moderate changes at PND23, showing that how microbes are acquired can influence the ongoing composition of the microbiome. The differences in the composition of the microbiome are modest; we did not perform a direct comparison between fecal and caecal microbiomes in this study, and the low biomass in the ileum may mean that modest changes may be more pronounced [47]. Future work that examines the differences in microbial content at different regions of the GI tract, in mucus, quantifies antimicrobial peptides, and measures pH between CS and VB will reinforce the data presented here [56].

We demonstrated that the mode of delivery influences gut barrier function and microbiome composition in early life (PND7) and that GOS/FOS and *B. breve* impact the microbiome during adolescence (PND23). Our previous work also showed that CS birth affects social and cognitive behavior [34]. Additionally, pre‐ and probiotic interventions mitigate microbiome alterations in CS mice. Since both the brain and microbiome undergo critical postnatal development, we investigated whether the link between impaired barrier function and microbiome changes in CS‐born mice (PND7) also affects the brain by measuring the expression of key neurotransmitter genes in the amygdala, a region sensitive to microbiome variations and probiotic interventions [13, 57, 58].

Tight junctions regulate epithelial permeability and several studies have investigated the positive influence that probiotics may have on epithelial permeability by modifying tight junction proteins. Given the importance of the intestinal barrier, it is not surprising that targeting the intestinal microbiome with pre‐ and probiotics is considered a modifiable target. Indeed, the commensal strain *Lactobacillus rhamnosus GG* induces development of barrier function via Cldn3 [59], whereas *Lactobacillus plantarum ZLP001* positively influenced tight junction expression through modulation of the microbiome [60]. Bifidobacterium, a prominent beneficial gut microbiota genus, is widely studied as a probiotic. In a mouse model of necrotising enterocolitis (NEC), *B. infantis* prevented an increase in intestinal permeability and maintained Claudin 4 and Occludin localization at tight junction structures, highlighting the potential of probiotic interventions.

This study focussed solely on male mice; future studies should examine female mice. Also, not measuring tight junction proteins as opposed to tight‐junction gene expression represents another limitation of this study. In addition, using an outbred strain may explain the high variability in some of the results. Future work should address the translational potential of the data presented here and consider if the mechanisms presented are also amenable to pre‐ and probiotic intervention at other physiological barriers, such as the blood–brain barrier and the skin.

## Author Contributions

Study design: A.P.V.S., I.B.R., J.K., K.R., T.D., and J.F.C. Data collection: A.P.V.S., A.M.S., M.L., and A.G. Data analysis: A.P.V.S., G.M.M., A.G., and T.B. Data interpretation: A.P.V.S., G.M.M., A.M.S., M.L., T.B., K.R., P.F., A.G., and M.R.‐A. Manuscript drafting: A.P.V.S., T.B., and G.M.M. Manuscript review: G.M.M., A.P.V.S., I.B.R., J.K., and J.F.C. All authors approved the final draft of the manuscript.

## Conflicts of Interest

J.F.C. is funded by the Science Foundation Ireland (SFI/12/RC/2273_P2), Saks Kavanaugh Foundation and Swiss National Science Foundation project CRSII5_186346/NMS2068 and has received research funding from IFF, Reckitt and Nutricia; he has been an invited speaker at meetings organized by Freisland Campina and Nutricia and has served as a consultant for Nestle.

## Supporting information


Table S1.

Table S2.

Table S3.

Table S4.

Table S5.

Table S6.

Data S1.

Data S2.

Data S3.

## Data Availability

The data that support the findings of this study are openly available in ENA at https://www.ebi.ac.uk/ena/browser/home, reference number PRJEB81708.
