# Supplementary material for: Targeting the Microbiota Reverses C‐Section‐Induced Effects on Intestinal Permeability, Microbiota Composition, and Amygdala Gene Expression in the Mouse
Source: Neurogastroenterol Motil. 2025 Jun 26;37(12):e70107. doi: 10.1111/nmo.70107 (PMC12623274; doi:10.1111/nmo.70107)
Supplement: Supplementary file 1 — Table S1. [file NMO-37-e70107-s002.docx]

**Supplementary Table 1: Alpha diversity of the Intestinal Microbiome is not affected by Mode of Delivery nor intervention at PND7 and PND23.**

| **Group** | **Chao1** | **Asymptotic Simpson** | **Asymptotic**  **Shannon** |
| --- | --- | --- | --- |
| **Postnatal Day 7** | | | |
| **Natural Born** | 10.20 ±0.47 | 0.28±0.07 | 0.54±0.11 |
| **Caesarean Section** | 11.63±1.70 | 0.39±0.04 | 0.73±0.07 |
| **Caesarean Section (Bif Breve)** | 9.88±0.95 | 0.40±0.03 | 0.67±0.04 |
| **Caesarean Section (Gos/Fos)** | 11.11±0.93 | 0.31±0.07 | 0.57±0.12 |
| **Postnatal Day 23** | | | |
| **Natural Born** | 32.71±1.06 | 0.79±0.02 | 2.11±0.09 |
| **Caesarean Section** | 31.88±3.47 | 0.83±0.01 | 2.25±0.13 |
| **Caesarean Section (Bif Breve)** | 30.71±0.61 | 0.83±0.01 | 2.22±0.08 |
| **Caesarean Section (Gos/Fos)** | 24.09±2.63 | 0.61±0.02 | 1.67±0.11 |

Alpha diversity indices of ileal microbiota composition at PND 7 (n = 10, 8, 8, 9 animals/group at PND7) and PND23 (n = 7, 8, 7, 8 animals/group at PND23). Values presented as mean ± SEM of the relevant alpha diversity metric.
