# Supplementary material for: Targeting the Microbiota Reverses C‐Section‐Induced Effects on Intestinal Permeability, Microbiota Composition, and Amygdala Gene Expression in the Mouse
Source: Neurogastroenterol Motil. 2025 Jun 26;37(12):e70107. doi: 10.1111/nmo.70107 (PMC12623274; doi:10.1111/nmo.70107)
Supplement: Supplementary file 2 — Table S2. [file NMO-37-e70107-s005.docx]

**Supplementary Table 2A**

**EF AIN 93G Control Diet (S9262-E360) 10mm Casein >> sterilized 25 kGy**

| **Crude Nutrients** | **%** | **Additives** | **per Kg** |
| --- | --- | --- | --- |
| Crude Protein | 17.6 | Vitamin A [IU / IE] | 4000 |
| Crude fat | 7.1 | Vitamin D3 [IU / IE] | 1000 |
| Crude fibre | 5 | Vitamin E [mg] | 75 |
| Crude ash | 3.1 | Vitamin K3 [mg] | 4 |
| Starch | 38.2 | Vitamin C [mg] | - |
| Sugar | 11.2 | Copper [mg] | 11 |

**Energy 16.2 MJ [or kcal] ME/kg**

**Supplementary Table 2B**

**EF AIN 93G GF/ 6% GOS, 0.3% FOS (S9262-E364) 10mm Casein >> sterilized 25 kGy**

| **Crude Nutrients** | **%** |  | **Additives** | **per Kg** |
| --- | --- | --- | --- | --- |
| Crude Protein | 17.6 |  | Vitamin A [IU / IE] | 4000 |
| Crude fat | 7.1 |  | Vitamin D3 [IU / IE] | 1000 |
| Crude fibre | 5 |  | Vitamin E [mg] | 75 |
| Crude ash | 3.1 |  | Vitamin K3 [mg] | 4 |
| Starch | 38.2 |  | Vitamin C [mg] | - |
| Sugar | 11.2 |  | Copper [mg] | 11 |

**Energy 16.2 MJ [or kcal] ME/kg**
