# Supplementary material for: Targeting the Microbiota Reverses C‐Section‐Induced Effects on Intestinal Permeability, Microbiota Composition, and Amygdala Gene Expression in the Mouse
Source: Neurogastroenterol Motil. 2025 Jun 26;37(12):e70107. doi: 10.1111/nmo.70107 (PMC12623274; doi:10.1111/nmo.70107)
Supplement: Supplementary file 4 — Table S4. [file NMO-37-e70107-s004.docx]

**Supplementary Table 4 Animal Timepoint Numbers**

|  | **Vaginally Born** | **C-section** | **C-section B. breve** | **C-section GOS/FOS** |
| --- | --- | --- | --- | --- |
| **Plasma FITC** | | | | |
| FITC p7 | 10 (1) **(5)** | 10 **(4)** | 10 **(6)** | 10(1) **(5)** |
| FITC p14 | 10 **(7)** | 10 **(5)** | 10 **(6)** | 10 **(5)** |
| FITC p23 | 9 **(5)** | 9 **(4)** | 10(2) **(4)** | 9 **(4)** |
| FITC p35 | 10 **(6)** | 9 **(5)** | 9 **(4)** | 9 **(3)** |
| **Ileal PCR** | | | | |
| Tjp p7 | 8(1) | 10 | 8(1) | 10 |
| Occludin p7 | 10 | 10(1) | 8 | 10 |
| Epcam p7 | 10 | 10 | 10 | 8 |
| Claudin3 p7 | 10 | 10 | 10(1) | 8 |
| Occludin p14 | 6 | 10 | 10 | 10 |
| Tjp p14 | 6 | 10 | 10 | 10 |
| Epcam p14 | 6 | 10 | 10 | 10 |
| Claudin3 p14 | 6 | 10 | 10 | 10 (1) |
| **Microbiome** | | | | |
| PND7 | 10 | 8 | 8 | 9 |
| PND23 | 7 | 8 | 7 | 8 |
| **Amygdala PCR** | | | | |
| Gabra2 | 10 | 10 | 7 | 8 |
| Gabbr1 | 10 | 10 | 7 | 8 |
| Grin2a | 10 | 10 | 7 | 8 |
| Grin2b | 10 | 10 (2) | 7 | 8 |
| Nr3c1 | 10 | 10 | 7 | 8 |
| Nr3c2 | 10 | 10 | 7 | 8 |
| Bdnf | 10 | 10 | 7 | 8 |
| Slc6a4 | 10 (1) | 10 (2) | 7 | 8 |
| Tph | 10 | 10 (1) | 7 | 8 |

Data presented as the number of animals used at each timepoint for each experiment. The number in brackets represent technical outliers that were removed from the analysis. Data in bold parentheses represents the number of litters that animals in that group were randomly chosen from.
