# Supplementary material for: Targeting the Microbiota Reverses C‐Section‐Induced Effects on Intestinal Permeability, Microbiota Composition, and Amygdala Gene Expression in the Mouse
Source: Neurogastroenterol Motil. 2025 Jun 26;37(12):e70107. doi: 10.1111/nmo.70107 (PMC12623274; doi:10.1111/nmo.70107)
Supplement: Supplementary file 7 — Data S1. [file NMO-37-e70107-s001.docx]

**Supplementary Table 1: Alpha diversity of the Intestinal Microbiome is not affected by Mode of Delivery nor intervention at PND7 and PND23.**

| **Group** | **Chao1** | **Asymptotic Simpson** | **Asymptotic**  **Shannon** |
| --- | --- | --- | --- |
| **Postnatal Day 7** | | | |
| **Natural Born** | 10.20 ±0.47 | 0.28±0.07 | 0.54±0.11 |
| **Caesarean Section** | 11.63±1.70 | 0.39±0.04 | 0.73±0.07 |
| **Caesarean Section (Bif Breve)** | 9.88±0.95 | 0.40±0.03 | 0.67±0.04 |
| **Caesarean Section (Gos/Fos)** | 11.11±0.93 | 0.31±0.07 | 0.57±0.12 |
| **Postnatal Day 23** | | | |
| **Natural Born** | 32.71±1.06 | 0.79±0.02 | 2.11±0.09 |
| **Caesarean Section** | 31.88±3.47 | 0.83±0.01 | 2.25±0.13 |
| **Caesarean Section (Bif Breve)** | 30.71±0.61 | 0.83±0.01 | 2.22±0.08 |
| **Caesarean Section (Gos/Fos)** | 24.09±2.63 | 0.61±0.02 | 1.67±0.11 |

Alpha diversity indices of ileal microbiota composition at PND 7 (n = 10, 8, 8, 9 animals/group at PND7) and PND23 (n = 7, 8, 7, 8 animals/group at PND23). Values presented as mean ± SEM of the relevant alpha diversity metric.

**Supplementary Table 2A**

**EF AIN 93G Control Diet (S9262-E360) 10mm Casein >> sterilized 25 kGy**

| **Crude Nutrients** | **%** | **Additives** | **per Kg** |
| --- | --- | --- | --- |
| Crude Protein | 17.6 | Vitamin A [IU / IE] | 4000 |
| Crude fat | 7.1 | Vitamin D3 [IU / IE] | 1000 |
| Crude fibre | 5 | Vitamin E [mg] | 75 |
| Crude ash | 3.1 | Vitamin K3 [mg] | 4 |
| Starch | 38.2 | Vitamin C [mg] | - |
| Sugar | 11.2 | Copper [mg] | 11 |

**Energy 16.2 MJ [or kcal] ME/kg**

**Supplementary Table 2B**

**EF AIN 93G GF/ 6% GOS, 0.3% FOS (S9262-E364) 10mm Casein >> sterilized 25 kGy**

| **Crude Nutrients** | **%** |  | **Additives** | **per Kg** |
| --- | --- | --- | --- | --- |
| Crude Protein | 17.6 |  | Vitamin A [IU / IE] | 4000 |
| Crude fat | 7.1 |  | Vitamin D3 [IU / IE] | 1000 |
| Crude fibre | 5 |  | Vitamin E [mg] | 75 |
| Crude ash | 3.1 |  | Vitamin K3 [mg] | 4 |
| Starch | 38.2 |  | Vitamin C [mg] | - |
| Sugar | 11.2 |  | Copper [mg] | 11 |

**Energy 16.2 MJ [or kcal] ME/kg**

**Supplementary Table 3 – Arrive Guidelines**

| **Item** | **Recommendation** | **Section or Line Number** |
| --- | --- | --- |
| Study Design | The groups being compared, including control groups. If no control group has been used, the rationale should be stated. | There were 4 timepoints in the study, p7, p14, p21 and p35. At each timepoint there were 4 groups, 1. Vaginally born mice, 2. C-Section born mice, 3. C-Section born mice receiving B. breve and 4. C-Section born mice receiving GOS/FOS. The initial comparison was between vaginally born mice with c-section mice at p7, p14, p21 and p35 for intestinal permeability, microbiome composition, barrier function genes in the intestine and neuroplasticity genes in the amygdala. Subsequently we compared the effect of 2 dietary interventions (B. breve and GOS/FOS) on these outputs at the same timepoints in c-section mice. |
|  | The experimental unit | A single animal |
| Sample Size | Specify the exact number of experimental units allocated to each group, and the total number in each experiment. Also indicate the total number of animals used. | 10 individual mice were allocated to each group at each timepoint. The total number of mice used in each experiment, in each group and at each timepoint is in Supplementary Table 4. The total number of mice allocated to this experiment was 160. |
|  | Explain how the sample size was decided. Provide details of any a priori sample size calculation, if done. | An examination of our own publications, in-house data, and a literature review were carried out to determine probable effect sizes a power analysis was conducted (α < 0.05, power = 80%) and numbers chosen are the minimum numbers required for statistical analysis for behavioural and physiological analysis (in this case permeability) in these experiments. Using the Software PS Power and Sample Size Calculation by Dupont and Plummer, a sample size of 10 animals per group is required to achieve an α of 0.05 and power of 80%. A review by Sauro et al. (2003) and publications in our research area, sample  Sauro MD, Jorgensen RS, Pedlow CT (2003) Stress, glucocorticoids, and memory: a meta-analytic review. Stress 6(4):235-45. |
| Inclusion and Exclusion Criteria | Describe any criteria used for including and excluding animals (or experimental units) during the experiment, and data points during the analysis. Specify if these criteria were established a priori. If no criteria were set, state this explicitly. | Animals were included in the study If they were visibly healthy, appropriate weight and of the right sex. Male mice were used in all experiments. Animals or experimental units were excluded if they experienced significant weight loss or visible illness. No specific criteria were set. |
|  | For each experimental group, report any animals, experimental units or data points not included in the analysis and explain why. If there were no exclusions, state so. | See table 4 (below).  Extreme outliers were excluded when values exceeded 2 x Standard Deviations from the mean.  For the microbiome analysis we excluded species that were only detected as non-zero in 5% or fewer of total samples from our count table as ratios are invariant to subsetting, and this study employs compositional data analysis techniques. |
|  | For each analysis, report the exact value of n in each experimental group. | See Table 4 |
| Randomisation | State whether randomisation was used to allocate experimental units to control and treatment groups. If done, provide the method used to generate the randomisation sequence. | Interventions were administered initially in a random fashion. Animals were randomly assigned to groups from 4-6 litters. |
|  | Describe the strategy used to minimise potential confounders such as the order of treatments and measurements, or animal/cage location. If confounders were not controlled, state this explicitly. | All treatments were performed in the same timed sequence on the same animal at the same time of day. E.g., animal 1 treated first, animal 40 treated last. All culls were done by the same people, and all lab-based analysis was done in a randomised fashion. |
| Blinding | Describe who was aware of the group allocation at the different stages of the experiment (during the allocation, the conduct of the experiment, the outcome assessment, and the data analysis). | The lead researcher (first author) was aware of the group allocation at the start of the experiment and at the very end for culling. All other analysis was done in a blinded manner by other researchers (FITC, PCR Microbiome). |
| Outcome measures | Clearly define all outcome measures assessed (e.g. cell death, molecular markers, or behavioural changes). | Intestinal permeability (FITC)  Gene expression (Ileum and brain).  Microbiome composition |
|  | For hypothesis-testing studies, specify the primary outcome measure, i.e. the outcome measure that was used to determine the sample size. | Intestinal permeability at p7 |

**Supplementary Table 4 Animal Timepoint Numbers**

|  | **Vaginally Born** | **C-section** | **C-section B. breve** | **C-section GOS/FOS** |
| --- | --- | --- | --- | --- |
| **Plasma FITC** | | | | |
| FITC p7 | 10 (1) **(5)** | 10 **(4)** | 10 **(6)** | 10(1) **(5)** |
| FITC p14 | 10 **(7)** | 10 **(5)** | 10 **(6)** | 10 **(5)** |
| FITC p23 | 9 **(5)** | 9 **(4)** | 10(2) **(4)** | 9 **(4)** |
| FITC p35 | 10 **(6)** | 9 **(5)** | 9 **(4)** | 9 **(3)** |
| **Ileal PCR** | | | | |
| Tjp p7 | 8(1) | 10 | 8(1) | 10 |
| Occludin p7 | 10 | 10(1) | 8 | 10 |
| Epcam p7 | 10 | 10 | 10 | 8 |
| Claudin3 p7 | 10 | 10 | 10(1) | 8 |
| Occludin p14 | 6 | 10 | 10 | 10 |
| Tjp p14 | 6 | 10 | 10 | 10 |
| Epcam p14 | 6 | 10 | 10 | 10 |
| Claudin3 p14 | 6 | 10 | 10 | 10 (1) |
| **Microbiome** | | | | |
| PND7 | 10 | 8 | 8 | 9 |
| PND23 | 7 | 8 | 7 | 8 |
| **Amygdala PCR** | | | | |
| Gabra2 | 10 | 10 | 7 | 8 |
| Gabbr1 | 10 | 10 | 7 | 8 |
| Grin2a | 10 | 10 | 7 | 8 |
| Grin2b | 10 | 10 (2) | 7 | 8 |
| Nr3c1 | 10 | 10 | 7 | 8 |
| Nr3c2 | 10 | 10 | 7 | 8 |
| Bdnf | 10 | 10 | 7 | 8 |
| Slc6a4 | 10 (1) | 10 (2) | 7 | 8 |
| Tph | 10 | 10 (1) | 7 | 8 |

Data presented as the number of animals used at each timepoint for each experiment. The number in brackets represent technical outliers that were removed from the analysis. Data in bold parentheses represents the number of litters that animals in that group were randomly chosen from.
