# Supplementary material for: Targeting the Microbiota Reverses C‐Section‐Induced Effects on Intestinal Permeability, Microbiota Composition, and Amygdala Gene Expression in the Mouse
Source: Neurogastroenterol Motil. 2025 Jun 26;37(12):e70107. doi: 10.1111/nmo.70107 (PMC12623274; doi:10.1111/nmo.70107)
Supplement: Supplementary file 8 — Data S2. [file NMO-37-e70107-s003.pdf]

	Bacteria	anovas.gro	anovas.gro	anovas.gro	anovas.gro	anovas.gro	tukeys.CS	tukeys.CS
1	Bacteria_A	3	0.5	0.18	0.69	0.53	0.12	-0.69
2	Bacteria_A	3	2.55	4.05	0.07	12.15	-1.01	-2.72
3	Bacteria_A	3	0.18	0.21	0.91	0.63	0.32	-1.13
4	Bacteria_A	3	0.47	0.39	0.71	1.18	0.21	-1.03
5	Bacteria_A	3	1.06	0.01	0.38	0.04	-0.08	-0.23
6	Bacteria_A	3	1.06	0.01	0.38	0.04	-0.08	-0.23
7	Bacteria_A	3	1.06	0.01	0.38	0.04	-0.08	-0.23
8	Bacteria_B	3	1.65	0.53	0.2	1.59	-0.08	-0.85
9	Bacteria_B	3	1.06	0.01	0.38	0.04	-0.08	-0.23
10	Bacteria_B	3	1.06	0.01	0.38	0.04	-0.08	-0.23
11	Bacteria_B	3	1.06	0.01	0.38	0.04	-0.08	-0.23
12	Bacteria_B	3	1.06	0.01	0.38	0.04	-0.08	-0.23
13	Bacteria_B	3	1.06	0.01	0.38	0.04	-0.08	-0.23
14	Bacteria_B	3	0.66	0.05	0.58	0.16	0.14	-0.25
15	Bacteria_D	3	1.06	0.01	0.38	0.04	-0.08	-0.23
16	Bacteria_Fi	3	5.34	1.43	0	4.28	0.82	0.12
17	Bacteria_Fi	3	0.19	1.82	0.9	5.45	1.17	-3.07
18	Bacteria_Fi	3	0.55	4.38	0.65	13.14	-1.3	-5.11
19	Bacteria_Fi	3	8.42	30.41	0	91.23	2.31	-0.27
20	Bacteria_Fi	3	1.06	0.01	0.38	0.04	-0.08	-0.23
21	Bacteria_Fi	3	1.06	0.01	0.38	0.04	-0.08	-0.23
22	Bacteria_Fi	3	1.06	0.01	0.38	0.04	-0.08	-0.23
23	Bacteria_Fi	3	1.06	0.01	0.38	0.04	-0.08	-0.23
24	Bacteria_Fi	3	1.06	0.01	0.38	0.04	-0.08	-0.23
25	Bacteria_Fi	3	0.67	0.01	0.58	0.04	-0.08	-0.27
26	Bacteria_Fi	3	1.06	0.01	0.38	0.04	-0.08	-0.23
27	Bacteria_Fi	3	1.06	0.01	0.38	0.04	-0.08	-0.23
28	Bacteria_Fi	3	1.06	0.01	0.38	0.04	-0.08	-0.23
29	Bacteria_Fi	3	1.06	0.01	0.38	0.04	-0.08	-0.23
30	Bacteria_Fi	3	1.06	0.01	0.38	0.04	-0.08	-0.23
31	Bacteria_Fi	3	1.13	1.41	0.35	4.23	-0.9	-2.42
32	Bacteria_Fi	3	1.06	0.01	0.38	0.04	-0.08	-0.23
33	Bacteria_Fi	3	1.06	0.01	0.38	0.04	-0.08	-0.23
34	Bacteria_Fi	3	1.06	0.01	0.38	0.04	-0.08	-0.23
35	Bacteria_Fi	3	1.06	0.01	0.38	0.04	-0.08	-0.23
36	Bacteria_Fi	3	1.06	0.01	0.38	0.04	-0.08	-0.23
37	Bacteria_Fi	3	1.06	0.01	0.38	0.04	-0.08	-0.23
38	Bacteria_Fi	3	1.06	0.01	0.38	0.04	-0.08	-0.23
39	Bacteria_Fi	3	1.06	0.01	0.38	0.04	-0.08	-0.23
40	Bacteria_Fi	3	1.06	0.01	0.38	0.04	-0.08	-0.23
41	Bacteria_Fi	3	1.06	0.01	0.38	0.04	-0.08	-0.23
42	Bacteria_Fi	3	1.06	0.01	0.38	0.04	-0.08	-0.23
43	Bacteria_Fi	3	1.06	0.01	0.38	0.04	-0.08	-0.23
44	Bacteria_Fi	3	1.06	0.01	0.38	0.04	-0.08	-0.23
45	Bacteria_Fi	3	1.06	0.01	0.38	0.04	-0.08	-0.23
46	Bacteria_Fi	3	1.06	0.01	0.38	0.04	-0.08	-0.23
47	Bacteria_Fi	3	1.06	0.01	0.38	0.04	-0.08	-0.23
48	Bacteria_Fi	3	1.06	0.01	0.38	0.04	-0.08	-0.23
49	Bacteria_Fi	3	1.06	0.01	0.38	0.04	-0.08	-0.23
50	Bacteria_P	3	0.28	0.08	0.84	0.23	0.18	-0.53
51	Bacteria_P	3	0.88	0.71	0.46	2.14	0.12	-1.11
52	Bacteria_P	3	0.12	0.08	0.95	0.24	0.17	-0.93
53	Bacteria_P	3	1.06	0.01	0.38	0.04	-0.08	-0.23
54	Bacteria_P	3	1.06	0.01	0.38	0.04	-0.08	-0.23
55	Bacteria_P	3	1.06	0.01	0.38	0.04	-0.08	-0.23

56 Bacteria_P	3	1.06	0.01	0.38	0.04	-0.08	-0.23
57 Bacteria_P	3	4.98	47.55	0.01	142.66	3.09	-1.11
58 Bacteria_P	3	1.34	17.47	0.28	52.4	-1.31	-6.2
59 Bacteria_P	3	1.06	0.01	0.38	0.04	-0.08	-0.23
60 Bacteria_P	3	1	0.92	0.41	2.75	0.23	-1.07
61 Bacteria_V	3	0.93	0.09	0.44	0.28	-0.08	-0.51
62 Unknown_U	3	0.88	0.94	0.46	2.82	-0.75	-2.15

tukeys.CS(	tukeys.CS(	tukeys.CS(	tukeys.CS(	tukeys.CS(	tukeys.CS(	tukeys.CS(	tukeys.CS(	tukeys.CS(
0.98	0.93	-0.04	-0.83	1	0.74	-0.16	-0.95	0.94
0.39	0.7	0.08	-1.58	1	1.74	1.09	-0.57	0.3
0.93	1.78	0.35	-1.06	0.91	1.77	0.03	-1.39	1
0.97	1.46	0.28	-0.93	0.92	1.49	0.07	-1.14	1
0.46	0.07	-0.04	-0.19	0.85	0.1	0.04	-0.11	0.89
0.46	0.07	-0.04	-0.19	0.85	0.1	0.04	-0.11	0.89
0.46	0.07	-0.04	-0.19	0.85	0.1	0.04	-0.11	0.89
0.99	0.69	0.44	-0.31	0.4	1.19	0.52	-0.23	0.26
0.46	0.07	-0.04	-0.19	0.85	0.1	0.04	-0.11	0.89
0.46	0.07	-0.04	-0.19	0.85	0.1	0.04	-0.11	0.89
0.46	0.07	-0.04	-0.19	0.85	0.1	0.04	-0.11	0.89
0.46	0.07	-0.04	-0.19	0.85	0.1	0.04	-0.11	0.89
0.46	0.07	-0.04	-0.19	0.85	0.1	0.04	-0.11	0.89
0.77	0.53	0.13	-0.24	0.78	0.51	-0.01	-0.38	1
0.46	0.07	-0.04	-0.19	0.85	0.1	0.04	-0.11	0.89
0.02	1.52	-0.04	-0.72	1	0.64	-0.86	-1.54	0.01
0.88	5.41	0.58	-3.54	0.98	4.7	-0.59	-4.71	0.98
0.79	2.52	-1.24	-4.95	0.8	2.48	0.06	-3.65	1
0.09	4.89	3.82	1.31	0	6.33	1.51	-1	0.38
0.46	0.07	-0.04	-0.19	0.85	0.1	0.04	-0.11	0.89
0.46	0.07	-0.04	-0.19	0.85	0.1	0.04	-0.11	0.89
0.46	0.07	-0.04	-0.19	0.85	0.1	0.04	-0.11	0.89
0.46	0.07	-0.04	-0.19	0.85	0.1	0.04	-0.11	0.89
0.46	0.07	-0.04	-0.19	0.85	0.1	0.04	-0.11	0.89
0.68	0.11	0.01	-0.18	1	0.19	0.09	-0.1	0.62
0.46	0.07	-0.04	-0.19	0.85	0.1	0.04	-0.11	0.89
0.46	0.07	-0.04	-0.19	0.85	0.1	0.04	-0.11	0.89
0.46	0.07	-0.04	-0.19	0.85	0.1	0.04	-0.11	0.89
0.46	0.07	-0.04	-0.19	0.85	0.1	0.04	-0.11	0.89
0.46	0.07	-0.04	-0.19	0.85	0.1	0.04	-0.11	0.89
0.39	0.61	-0.86	-2.34	0.4	0.61	0.04	-1.44	1
0.46	0.07	-0.04	-0.19	0.85	0.1	0.04	-0.11	0.89
0.46	0.07	-0.04	-0.19	0.85	0.1	0.04	-0.11	0.89
0.46	0.07	-0.04	-0.19	0.85	0.1	0.04	-0.11	0.89
0.46	0.07	-0.04	-0.19	0.85	0.1	0.04	-0.11	0.89
0.46	0.07	-0.04	-0.19	0.85	0.1	0.04	-0.11	0.89
0.46	0.07	-0.04	-0.19	0.85	0.1	0.04	-0.11	0.89
0.46	0.07	-0.04	-0.19	0.85	0.1	0.04	-0.11	0.89
0.46	0.07	-0.04	-0.19	0.85	0.1	0.04	-0.11	0.89
0.46	0.07	-0.04	-0.19	0.85	0.1	0.04	-0.11	0.89
0.46	0.07	-0.04	-0.19	0.85	0.1	0.04	-0.11	0.89
0.46	0.07	-0.04	-0.19	0.85	0.1	0.04	-0.11	0.89
0.46	0.07	-0.04	-0.19	0.85	0.1	0.04	-0.11	0.89
0.46	0.07	-0.04	-0.19	0.85	0.1	0.04	-0.11	0.89
0.46	0.07	-0.04	-0.19	0.85	0.1	0.04	-0.11	0.89
0.46	0.07	-0.04	-0.19	0.85	0.1	0.04	-0.11	0.89
0.46	0.07	-0.04	-0.19	0.85	0.1	0.04	-0.11	0.89
0.46	0.07	-0.04	-0.19	0.85	0.1	0.04	-0.11	0.89
0.46	0.07	-0.04	-0.19	0.85	0.1	0.04	-0.11	0.89
0.91	0.88	0.06	-0.63	1	0.74	-0.12	-0.81	0.96
0.99	1.34	0.62	-0.57	0.5	1.81	0.5	-0.69	0.66
0.97	1.27	-0.05	-1.11	1	1.02	-0.22	-1.29	0.94
0.46	0.07	-0.04	-0.19	0.85	0.1	0.04	-0.11	0.89
0.46	0.07	-0.04	-0.19	0.85	0.1	0.04	-0.11	0.89
0.46	0.07	-0.04	-0.19	0.85	0.1	0.04	-0.11	0.89

0.46	0.07	-0.04	-0.19	0.85	0.1	0.04	-0.11	0.89
0.21	7.28	0.15	-3.93	1	4.23	-2.94	-7.01	0.23
0.89	3.59	-3.01	-7.77	0.33	1.75	-1.71	-6.47	0.76
0.46	0.07	-0.04	-0.19	0.85	0.1	0.04	-0.11	0.89
0.96	1.53	0.73	-0.53	0.41	2	0.51	-0.76	0.7
0.96	0.35	0.17	-0.25	0.7	0.59	0.25	-0.17	0.4
0.48	0.65	-0.37	-1.73	0.88	0.99	0.38	-0.98	0.87

tukeys.CS(	tukeys.NB(	tukeys.NB(	tukeys.NB(	tukeys.NB(	tukeys.NB(	tukeys.NB(	tukeys.NB(	tukeys.NB(
0.62	-0.22	-0.99	0.86	0.55	-0.34	-1.1	0.64	0.43
2.75	0.63	-0.99	0.72	2.25	1.64	0.02	0.05	3.26
1.44	0.23	-1.15	0.97	1.62	-0.09	-1.47	1	1.29
1.28	-0.17	-1.35	0.98	1.01	-0.38	-1.56	0.81	0.8
0.18	0	-0.14	1	0.14	0.08	-0.06	0.43	0.22
0.18	0	-0.14	1	0.14	0.08	-0.06	0.43	0.22
0.18	0	-0.14	1	0.14	0.08	-0.06	0.43	0.22
1.27	-0.06	-0.78	1	0.67	0.03	-0.7	1	0.75
0.18	0	-0.14	1	0.14	0.08	-0.06	0.43	0.22
0.18	0	-0.14	1	0.14	0.08	-0.06	0.43	0.22
0.18	0	-0.14	1	0.14	0.08	-0.06	0.43	0.22
0.18	0	-0.14	1	0.14	0.08	-0.06	0.43	0.22
0.18	0	-0.14	1	0.14	0.08	-0.06	0.43	0.22
0.37	0	-0.37	1	0.37	-0.14	-0.51	0.73	0.23
0.18	0	-0.14	1	0.14	0.08	-0.06	0.43	0.22
-0.18	0	-0.67	1	0.66	-0.82	-1.48	0.01	-0.15
3.53	0.56	-3.46	0.98	4.58	-0.61	-4.63	0.98	3.41
3.77	-0.07	-3.69	1	3.55	1.22	-2.4	0.8	4.85
4.01	4.13	1.68	0	6.58	1.82	-0.63	0.2	4.26
0.18	0	-0.14	1	0.14	0.08	-0.06	0.43	0.22
0.18	0	-0.14	1	0.14	0.08	-0.06	0.43	0.22
0.18	0	-0.14	1	0.14	0.08	-0.06	0.43	0.22
0.18	0	-0.14	1	0.14	0.08	-0.06	0.43	0.22
0.18	0	-0.14	1	0.14	0.08	-0.06	0.43	0.22
0.27	0	-0.19	1	0.18	0.08	-0.11	0.65	0.26
0.18	0	-0.14	1	0.14	0.08	-0.06	0.43	0.22
0.18	0	-0.14	1	0.14	0.08	-0.06	0.43	0.22
0.18	0	-0.14	1	0.14	0.08	-0.06	0.43	0.22
0.18	0	-0.14	1	0.14	0.08	-0.06	0.43	0.22
0.18	0	-0.14	1	0.14	0.08	-0.06	0.43	0.22
1.51	-0.63	-2.07	0.64	0.81	0.27	-1.17	0.96	1.71
0.18	0	-0.14	1	0.14	0.08	-0.06	0.43	0.22
0.18	0	-0.14	1	0.14	0.08	-0.06	0.43	0.22
0.18	0	-0.14	1	0.14	0.08	-0.06	0.43	0.22
0.18	0	-0.14	1	0.14	0.08	-0.06	0.43	0.22
0.18	0	-0.14	1	0.14	0.08	-0.06	0.43	0.22
0.18	0	-0.14	1	0.14	0.08	-0.06	0.43	0.22
0.18	0	-0.14	1	0.14	0.08	-0.06	0.43	0.22
0.18	0	-0.14	1	0.14	0.08	-0.06	0.43	0.22
0.18	0	-0.14	1	0.14	0.08	-0.06	0.43	0.22
0.18	0	-0.14	1	0.14	0.08	-0.06	0.43	0.22
0.18	0	-0.14	1	0.14	0.08	-0.06	0.43	0.22
0.18	0	-0.14	1	0.14	0.08	-0.06	0.43	0.22
0.18	0	-0.14	1	0.14	0.08	-0.06	0.43	0.22
0.18	0	-0.14	1	0.14	0.08	-0.06	0.43	0.22
0.18	0	-0.14	1	0.14	0.08	-0.06	0.43	0.22
0.18	0	-0.14	1	0.14	0.08	-0.06	0.43	0.22
0.18	0	-0.14	1	0.14	0.08	-0.06	0.43	0.22
0.18	0	-0.14	1	0.14	0.08	-0.06	0.43	0.22
0.57	0.19	-0.48	0.86	0.86	0.02	-0.65	1	0.69
1.69	0.46	-0.7	0.71	1.62	0.34	-0.82	0.86	1.5
0.85	0	-1.05	1	1.04	-0.18	-1.22	0.97	0.86
0.18	0	-0.14	1	0.14	0.08	-0.06	0.43	0.22
0.18	0	-0.14	1	0.14	0.08	-0.06	0.43	0.22
0.18	0	-0.14	1	0.14	0.08	-0.06	0.43	0.22

0.18	0	-0.14	1	0.14	0.08	-0.06	0.43	0.22
1.14	-2.57	-6.55	0.31	1.41	-5.66	-9.64	0	-1.68
3.05	-2.88	-7.52	0.35	1.77	-1.57	-6.22	0.8	3.08
0.18	0	-0.14	1	0.14	0.08	-0.06	0.43	0.22
1.77	0.55	-0.68	0.63	1.78	0.32	-0.91	0.89	1.55
0.67	0	-0.41	1	0.41	0.08	-0.33	0.95	0.49
1.74	-0.09	-1.42	1	1.24	0.66	-0.67	0.54	1.99

tukeys.NB(	tukeys.NB(	tukeys.NB(	tukeys.NB(	anovas.group Pr(>F).BH
-0.17	-0.92	0.92	0.57	0.75
0.55	-1.02	0.78	2.12	0.48
-0.12	-1.46	1	1.22	0.92
-0.45	-1.59	0.71	0.69	0.75
0.04	-0.09	0.84	0.18	0.48
0.04	-0.09	0.84	0.18	0.48
0.04	-0.09	0.84	0.18	0.48
-0.49	-1.2	0.25	0.21	0.48
0.04	-0.09	0.84	0.18	0.48
0.04	-0.09	0.84	0.18	0.48
0.04	-0.09	0.84	0.18	0.48
0.04	-0.09	0.84	0.18	0.48
0.04	-0.09	0.84	0.18	0.48
-0.13	-0.49	0.74	0.22	0.66
0.04	-0.09	0.84	0.18	0.48
0.04	-0.6	1	0.69	0.13
-0.02	-3.92	1	3.87	0.92
1.16	-2.35	0.8	4.67	0.72
0.31	-2.06	0.98	2.68	0.02
0.04	-0.09	0.84	0.18	0.48
0.04	-0.09	0.84	0.18	0.48
0.04	-0.09	0.84	0.18	0.48
0.04	-0.09	0.84	0.18	0.48
0.04	-0.09	0.84	0.18	0.48
-0.01	-0.18	1	0.17	0.66
0.04	-0.09	0.84	0.18	0.48
0.04	-0.09	0.84	0.18	0.48
0.04	-0.09	0.84	0.18	0.48
0.04	-0.09	0.84	0.18	0.48
0.04	-0.09	0.84	0.18	0.48
0.23	-1.16	0.97	1.62	0.48
0.04	-0.09	0.84	0.18	0.48
0.04	-0.09	0.84	0.18	0.48
0.04	-0.09	0.84	0.18	0.48
0.04	-0.09	0.84	0.18	0.48
0.04	-0.09	0.84	0.18	0.48
0.04	-0.09	0.84	0.18	0.48
0.04	-0.09	0.84	0.18	0.48
0.04	-0.09	0.84	0.18	0.48
0.04	-0.09	0.84	0.18	0.48
0.04	-0.09	0.84	0.18	0.48
0.04	-0.09	0.84	0.18	0.48
0.04	-0.09	0.84	0.18	0.48
0.04	-0.09	0.84	0.18	0.48
0.04	-0.09	0.84	0.18	0.48
0.04	-0.09	0.84	0.18	0.48
0.04	-0.09	0.84	0.18	0.48
0.04	-0.09	0.84	0.18	0.48
0.04	-0.09	0.84	0.18	0.48
0.14	-0.51	0.94	0.78	0.88
-0.16	-1.29	0.98	0.96	0.54
0.04	-0.97	1	1.05	0.95
0.04	-0.09	0.84	0.18	0.48
0.04	-0.09	0.84	0.18	0.48
0.04	-0.09	0.84	0.18	0.48

0.04	-0.09	0.84	0.18	0.48
-2.72	-6.58	0.24	1.13	0.13
0.14	-4.36	1	4.64	0.48
0.04	-0.09	0.84	0.18	0.48
-0.18	-1.38	0.97	1.01	0.51
-0.17	-0.57	0.66	0.23	0.53
0.28	-1.01	0.94	1.57	0.54
