# Supplementary material for: Targeting the Microbiota Reverses C‐Section‐Induced Effects on Intestinal Permeability, Microbiota Composition, and Amygdala Gene Expression in the Mouse
Source: Neurogastroenterol Motil. 2025 Jun 26;37(12):e70107. doi: 10.1111/nmo.70107 (PMC12623274; doi:10.1111/nmo.70107)
Supplement: Supplementary file 9 — Data S3. [file NMO-37-e70107-s008.pdf]

	Bacteria	anovas.gro	anovas.gro	anovas.gro	anovas.gro	anovas.gro	tukeys.CS(	tukeys.CS(
1	Bacteria_A	3	16.05	30.61	0	91.82	-4.35	-6.31
2	Bacteria_A	3	3.83	0.68	0.02	2.04	0.07	-0.53
3	Bacteria_A	3	0.89	0.27	0.46	0.81	-0.05	-0.83
4	Bacteria_A	3	2.36	0.49	0.09	1.47	-0.05	-0.7
5	Bacteria_A	3	1.38	0.64	0.27	1.92	0.47	-0.5
6	Bacteria_A	3	14.52	7.21	0	21.62	0.07	-0.93
7	Bacteria_A	3	2.29	0.68	0.1	2.04	0.48	-0.3
8	Bacteria_B	3	7.16	2.72	0	8.15	-0.05	-0.92
9	Bacteria_B	3	0.38	0.7	0.77	2.09	-0.25	-2.16
10	Bacteria_B	3	8.11	18.83	0	56.49	-0.12	-2.28
11	Bacteria_B	3	1.01	1.97	0.4	5.91	-1.15	-3.13
12	Bacteria_B	3	0.65	1.64	0.59	4.93	0.54	-1.72
13	Bacteria_B	3	2.66	9.37	0.07	28.12	0.72	-1.94
14	Bacteria_B	3	3.83	0.68	0.02	2.04	0.07	-0.53
15	Bacteria_D	3	0.59	1.3	0.62	3.89	-0.34	-2.43
16	Bacteria_Fi	3	3.31	0.64	0.04	1.91	0.01	-0.61
17	Bacteria_Fi	3	2.56	18.58	0.08	55.75	3.08	-0.75
18	Bacteria_Fi	3	2.77	8.32	0.06	24.97	-1.74	-4.2
19	Bacteria_Fi	3	0.57	0.49	0.64	1.46	-0.27	-1.59
20	Bacteria_Fi	3	8.33	15.31	0	45.93	0.56	-1.36
21	Bacteria_Fi	3	0.98	2.22	0.42	6.67	-0.13	-2.27
22	Bacteria_Fi	3	11.43	2.21	0	6.63	0.07	-0.55
23	Bacteria_Fi	3	3.87	4.4	0.02	13.21	1.81	0.3
24	Bacteria_Fi	3	0.76	1.09	0.53	3.26	-0.26	-1.96
25	Bacteria_Fi	3	0.29	0.61	0.83	1.82	-0.63	-2.69
26	Bacteria_Fi	3	6.44	4.79	0	14.37	0.34	-0.88
27	Bacteria_Fi	3	6	13.94	0	41.82	-0.2	-2.37
28	Bacteria_Fi	3	1.84	3.58	0.16	10.74	0.15	-1.83
29	Bacteria_Fi	3	1.72	3.5	0.19	10.49	0.11	-1.91
30	Bacteria_Fi	3	0.65	1.82	0.59	5.47	0.39	-1.98
31	Bacteria_Fi	3	10.79	19.03	0	57.09	0.12	-1.76
32	Bacteria_Fi	3	1.09	1.27	0.37	3.82	-0.08	-1.61
33	Bacteria_Fi	3	1.61	1.11	0.21	3.32	0.87	-0.31
34	Bacteria_Fi	3	0.58	0.88	0.63	2.65	-0.64	-2.39
35	Bacteria_Fi	3	0.2	0.15	0.89	0.45	0.31	-0.91
36	Bacteria_Fi	3	4.14	5.87	0.02	17.61	0.09	-1.6
37	Bacteria_Fi	3	1.32	2.13	0.29	6.4	-0.26	-2.07
38	Bacteria_Fi	3	5.99	14.8	0	44.4	-0.76	-3
39	Bacteria_Fi	3	11.6	20.95	0	62.86	-0.58	-2.49
40	Bacteria_Fi	3	0.98	1.37	0.42	4.11	0.14	-1.54
41	Bacteria_Fi	3	1.72	1.15	0.19	3.44	-0.04	-1.2
42	Bacteria_Fi	3	1.56	1.57	0.22	4.71	0.98	-0.44
43	Bacteria_Fi	3	0.34	0.22	0.8	0.66	0.4	-0.74
44	Bacteria_Fi	3	1.81	0.55	0.17	1.66	-0.07	-0.86
45	Bacteria_Fi	3	0.99	0.93	0.41	2.78	-0.84	-2.22
46	Bacteria_Fi	3	2.64	2.62	0.07	7.85	1.22	-0.19
47	Bacteria_Fi	3	2.64	3.03	0.07	9.08	0.09	-1.43
48	Bacteria_Fi	3	5.3	12.87	0.01	38.61	0.55	-1.67
49	Bacteria_Fi	3	3.51	22.17	0.03	66.51	-4.04	-7.61
50	Bacteria_P	3	3.83	0.68	0.02	2.04	0.07	-0.53
51	Bacteria_P	3	2.66	0.52	0.07	1.57	0.26	-0.37
52	Bacteria_P	3	2.48	0.55	0.08	1.65	0.06	-0.61
53	Bacteria_P	3	3.06	2.53	0.05	7.59	1.19	-0.1
54	Bacteria_P	3	1.13	2.57	0.35	7.72	-0.21	-2.35
55	Bacteria_P	3	2.55	3.86	0.08	11.59	-0.69	-2.44

56	Bacteria_P	3	0.61	1.69	0.61	5.06	-0.59	-2.95
57	Bacteria_P	3	3.66	25.72	0.03	77.16	3.49	-0.27
58	Bacteria_P	3	2.97	0.57	0.05	1.72	0.07	-0.55
59	Bacteria_P	3	0.79	1.51	0.51	4.54	-0.07	-2.04
60	Bacteria_P	3	0.19	0.59	0.9	1.76	-0.26	-2.78
61	Bacteria_V	3	5.38	24.21	0.01	72.64	0.67	-2.35
62	Unknown_	3	0.91	2.05	0.45	6.15	-0.81	-2.94

tukeys.CS(	tukeys.CS(	tukeys.CS(	tukeys.CS(	tukeys.CS(	tukeys.CS(	tukeys.CS(	tukeys.CS(	tukeys.CS(
0	-2.39	-3.04	-5	0	-1.08	1.31	-0.59	0.25
0.99	0.67	0.61	0.01	0.05	1.21	0.54	-0.04	0.07
1	0.73	0.35	-0.43	0.62	1.13	0.4	-0.35	0.48
1	0.6	0.49	-0.16	0.19	1.14	0.54	-0.09	0.11
0.55	1.44	0.61	-0.36	0.33	1.58	0.14	-0.8	0.98
1	1.07	1.94	0.94	0	2.94	1.87	0.91	0
0.35	1.25	0.66	-0.11	0.11	1.44	0.19	-0.56	0.9
1	0.82	1.21	0.34	0	2.09	1.26	0.42	0
0.98	1.67	0.47	-1.45	0.91	2.38	0.71	-1.14	0.72
1	2.05	-3.1	-5.27	0	-0.94	-2.99	-5.08	0
0.4	0.83	-1.02	-3	0.51	0.96	0.13	-1.78	1
0.91	2.79	-0.08	-2.34	1	2.18	-0.62	-2.8	0.86
0.88	3.38	2.34	-0.32	0.1	5.01	1.62	-0.95	0.33
0.99	0.67	0.61	0.01	0.05	1.21	0.54	-0.04	0.07
0.97	1.76	0.54	-1.56	0.9	2.63	0.87	-1.16	0.64
1	0.63	0.55	-0.07	0.1	1.17	0.54	-0.06	0.09
0.15	6.9	3.42	-0.4	0.09	7.24	0.34	-3.35	0.99
0.24	0.72	0.68	-1.78	0.87	3.14	2.42	0.04	0.05
0.94	1.05	0.27	-1.04	0.94	1.59	0.54	-0.73	0.65
0.85	2.49	1.07	-0.85	0.43	3	0.51	-1.35	0.87
1	2.01	-1.19	-3.32	0.44	0.95	-1.05	-3.12	0.51
0.99	0.69	1.08	0.46	0	1.71	1.01	0.41	0
0.01	3.33	0.55	-0.97	0.76	2.06	-1.27	-2.73	0.11
0.97	1.44	-0.89	-2.58	0.49	0.81	-0.63	-2.27	0.72
0.84	1.44	-0.55	-2.62	0.88	1.51	0.08	-1.92	1
0.87	1.57	-1.45	-2.68	0.02	-0.23	-1.79	-2.98	0
0.99	1.96	-2.43	-4.6	0.02	-0.27	-2.23	-4.32	0.03
1	2.13	-0.98	-2.96	0.54	1	-1.13	-3.04	0.38
1	2.14	-1.35	-3.38	0.28	0.67	-1.47	-3.43	0.19
0.97	2.76	-0.15	-2.52	1	2.23	-0.54	-2.83	0.92
1	2.01	-3.14	-5.02	0	-1.25	-3.26	-5.08	0
1	1.45	-0.8	-2.33	0.49	0.73	-0.72	-2.2	0.55
0.2	2.05	0.64	-0.54	0.46	1.81	-0.23	-1.37	0.94
0.75	1.11	-0.72	-2.47	0.68	1.03	-0.08	-1.77	1
0.9	1.53	0.29	-0.92	0.91	1.51	-0.02	-1.19	1
1	1.78	-1.71	-3.4	0.05	-0.02	-1.8	-3.43	0.03
0.98	1.55	-1.11	-2.92	0.35	0.69	-0.85	-2.6	0.55
0.78	1.47	-2.73	-4.96	0.01	-0.5	-1.96	-4.12	0.08
0.84	1.33	-3.71	-5.62	0	-1.8	-3.13	-4.97	0
1	1.82	-0.46	-2.14	0.87	1.22	-0.6	-2.23	0.74
1	1.11	0.76	-0.4	0.3	1.91	0.8	-0.32	0.23
0.26	2.41	0.25	-1.17	0.96	1.68	-0.73	-2.1	0.48
0.77	1.55	0.2	-0.94	0.96	1.35	-0.2	-1.31	0.96
0.99	0.71	0.52	-0.26	0.29	1.31	0.59	-0.17	0.17
0.36	0.53	-0.3	-1.68	0.93	1.07	0.54	-0.79	0.68
0.11	2.64	0.61	-0.81	0.64	2.02	-0.62	-1.98	0.61
1	1.61	0.85	-0.67	0.43	2.37	0.76	-0.7	0.49
0.9	2.76	2.71	0.5	0.01	4.92	2.16	0.03	0.05
0.02	-0.47	-1.15	-4.72	0.81	2.42	2.89	-0.56	0.12
0.99	0.67	0.61	0.01	0.05	1.21	0.54	-0.04	0.07
0.66	0.89	0.61	-0.02	0.06	1.24	0.34	-0.26	0.42
0.99	0.73	0.55	-0.12	0.14	1.22	0.49	-0.16	0.19
0.08	2.48	0.66	-0.63	0.51	1.95	-0.53	-1.78	0.65
0.99	1.93	-1.31	-3.45	0.36	0.83	-1.1	-3.17	0.48
0.7	1.05	-0.1	-1.85	1	1.64	0.59	-1.1	0.77

0.9	1.77	0.11	-2.25	1	2.47	0.7	-1.58	0.84
0.08	7.26	4.3	0.54	0.02	8.06	0.81	-2.83	0.93
0.99	0.69	0.61	-0.02	0.06	1.23	0.54	-0.06	0.09
1	1.9	-0.55	-2.52	0.87	1.42	-0.48	-2.38	0.9
0.99	2.26	-0.53	-3.04	0.94	1.99	-0.27	-2.7	0.99
0.93	3.68	3.67	0.66	0.01	6.68	3	0.09	0.04
0.72	1.32	-0.84	-2.97	0.7	1.29	-0.03	-2.09	1

tukeys.CS(	tukeys.NB(	tukeys.NB(	tukeys.NB(	tukeys.NB(	tukeys.NB(	tukeys.NB(	tukeys.NB(	tukeys.NB(
3.2	-4.43	-6.45	0	-2.4	-0.08	-2.04	1	1.88
1.12	-0.01	-0.63	1	0.61	-0.08	-0.68	0.98	0.52
1.15	0	-0.81	1	0.8	0.05	-0.73	1	0.83
1.17	0.03	-0.64	1	0.7	0.08	-0.56	0.98	0.73
1.07	0.1	-0.9	0.99	1.09	-0.38	-1.34	0.71	0.59
2.84	-0.01	-1.04	1	1.03	-0.08	-1.08	1	0.92
0.93	0.15	-0.65	0.95	0.95	-0.32	-1.1	0.66	0.45
2.11	0.25	-0.66	0.88	1.15	0.3	-0.58	0.79	1.17
2.56	0.07	-1.91	1	2.05	0.32	-1.6	0.97	2.24
-0.9	0.11	-2.12	1	2.35	0.23	-1.93	0.99	2.4
2.04	-0.92	-2.97	0.61	1.12	0.22	-1.76	0.99	2.2
1.56	-0.61	-2.94	0.89	1.72	-1.14	-3.4	0.52	1.11
4.19	-0.01	-2.76	1	2.74	-0.73	-3.39	0.88	1.93
1.12	-0.01	-0.63	1	0.61	-0.08	-0.68	0.98	0.52
2.9	-0.32	-2.48	0.98	1.85	0.02	-2.08	1	2.12
1.14	-0.07	-0.71	0.99	0.57	-0.08	-0.7	0.99	0.54
4.04	1.4	-2.54	0.76	5.35	-1.67	-5.5	0.63	2.15
4.8	-0.54	-3.09	0.94	2	1.2	-1.26	0.55	3.66
1.81	0.23	-1.13	0.97	1.59	0.5	-0.81	0.72	1.82
2.37	3.35	1.37	0	5.34	2.79	0.87	0	4.72
1.01	-0.6	-2.8	0.88	1.61	-0.47	-2.6	0.93	1.67
1.62	-0.01	-0.65	1	0.64	-0.08	-0.7	0.99	0.55
0.2	0.86	-0.7	0.45	2.43	-0.95	-2.47	0.33	0.56
1.01	-0.52	-2.27	0.85	1.23	-0.26	-1.95	0.97	1.44
2.07	-0.27	-2.4	0.99	1.86	0.36	-1.71	0.96	2.42
-0.61	-0.33	-1.59	0.89	0.94	-0.67	-1.89	0.45	0.55
-0.14	0.66	-1.57	0.85	2.9	0.87	-1.3	0.69	3.03
0.78	0.66	-1.39	0.81	2.7	0.51	-1.47	0.9	2.49
0.49	-0.44	-2.53	0.94	1.66	-0.55	-2.58	0.88	1.48
1.76	-0.8	-3.26	0.8	1.65	-1.2	-3.57	0.52	1.18
-1.44	-0.24	-2.18	0.99	1.71	-0.36	-2.24	0.95	1.53
0.76	0.08	-1.5	1	1.66	0.16	-1.37	0.99	1.69
0.9	0.78	-0.44	0.32	1.99	-0.09	-1.27	1	1.09
1.61	-0.74	-2.54	0.68	1.07	-0.1	-1.84	1	1.65
1.16	0.23	-1.02	0.96	1.49	-0.08	-1.29	1	1.14
-0.16	-0.03	-1.78	1	1.71	-0.12	-1.81	1	1.57
0.89	-0.01	-1.87	1	1.86	0.25	-1.56	0.98	2.06
0.19	0.41	-1.9	0.96	2.71	1.17	-1.06	0.49	3.4
-1.29	-1.02	-2.99	0.5	0.95	-0.44	-2.35	0.92	1.47
1.02	0.57	-1.16	0.8	2.31	0.43	-1.25	0.89	2.11
1.92	0.03	-1.17	1	1.22	0.07	-1.09	1	1.23
0.65	0.06	-1.41	1	1.53	-0.92	-2.35	0.31	0.5
0.91	0.1	-1.08	0.99	1.29	-0.3	-1.44	0.89	0.85
1.35	0.19	-0.62	0.91	1.01	0.27	-0.52	0.79	1.05
1.87	-0.47	-1.89	0.8	0.95	0.37	-1.01	0.88	1.74
0.75	-0.01	-1.47	1	1.45	-1.23	-2.65	0.1	0.18
2.23	-0.7	-2.27	0.62	0.87	-0.79	-2.31	0.5	0.73
4.3	-0.01	-2.29	1	2.28	-0.55	-2.77	0.9	1.66
6.34	-2.07	-5.76	0.43	1.61	1.97	-1.6	0.45	5.54
1.12	-0.01	-0.63	1	0.61	-0.08	-0.68	0.98	0.52
0.95	0.13	-0.52	0.95	0.78	-0.14	-0.77	0.93	0.49
1.13	-0.01	-0.7	1	0.68	-0.07	-0.74	0.99	0.6
0.72	-0.01	-1.34	1	1.33	-1.2	-2.49	0.08	0.09
0.97	-0.41	-2.62	0.96	1.8	-0.2	-2.34	0.99	1.94
2.28	1.05	-0.76	0.4	2.85	1.74	-0.01	0.05	3.49

2.97	-0.89	-3.33	0.75	1.54	-0.31	-2.66	0.98	2.05
4.44	2.37	-1.52	0.36	6.25	-1.12	-4.89	0.84	2.64
1.14	0.25	-0.4	0.72	0.89	0.18	-0.45	0.86	0.8
1.42	-0.98	-3.02	0.56	1.05	-0.92	-2.89	0.59	1.05
2.16	0.1	-2.5	1	2.69	0.35	-2.16	0.98	2.87
5.91	3.11	0	0.05	6.22	2.44	-0.57	0.14	5.45
2.03	0.15	-2.05	1	2.35	0.96	-1.17	0.61	3.09

tukeys.NB(	tukeys.NB(	tukeys.NB(	tukeys.NB(	anovas.group Pr(>F).BH
-1.39	-3.35	0.24	0.58	0
-0.62	-1.21	0.04	-0.02	0.07
-0.35	-1.13	0.61	0.43	0.58
-0.46	-1.1	0.24	0.19	0.18
-0.51	-1.48	0.48	0.45	0.42
-1.95	-2.95	0	-0.95	0
-0.51	-1.28	0.29	0.26	0.19
-0.97	-1.84	0.03	-0.09	0.01
-0.39	-2.31	0.94	1.52	0.82
3.22	1.05	0	5.38	0
0.09	-1.89	1	2.07	0.55
-0.53	-2.78	0.92	1.73	0.69
-2.35	-5.01	0.1	0.31	0.16
-0.62	-1.21	0.04	-0.02	0.07
-0.85	-2.95	0.68	1.25	0.7
-0.62	-1.24	0.05	0.01	0.1
-2.02	-5.84	0.48	1.81	0.16
-1.22	-3.68	0.53	1.24	0.16
-0.04	-1.36	1	1.28	0.7
2.28	0.35	0.02	4.2	0
0.59	-1.55	0.87	2.73	0.55
-1.09	-1.72	0	-0.47	0
0.31	-1.2	0.94	1.83	0.07
0.37	-1.33	0.93	2.07	0.64
0.28	-1.78	0.98	2.34	0.86
1.12	-0.1	0.08	2.35	0.01
3.1	0.93	0	5.26	0.02
1.64	-0.34	0.13	3.62	0.3
0.92	-1.11	0.61	2.94	0.32
-0.66	-3.03	0.87	1.72	0.69
2.9	1.02	0	4.79	0
0.88	-0.65	0.41	2.41	0.53
0.14	-1.03	0.99	1.32	0.34
-0.02	-1.77	1	1.73	0.7
-0.06	-1.27	1	1.16	0.9
1.68	-0.02	0.05	3.37	0.07
1.11	-0.7	0.36	2.91	0.44
3.14	0.9	0	5.37	0.02
2.69	0.78	0	4.6	0
1.04	-0.64	0.35	2.72	0.55
-0.73	-1.89	0.33	0.43	0.32
-0.2	-1.62	0.98	1.23	0.35
-0.1	-1.24	1	1.05	0.84
-0.33	-1.11	0.67	0.46	0.3
-0.17	-1.55	0.99	1.2	0.55
-0.62	-2.03	0.63	0.8	0.16
-1.55	-3.07	0.04	-0.03	0.16
-2.72	-4.93	0.01	-0.5	0.03
-0.92	-4.49	0.89	2.65	0.09
-0.62	-1.21	0.04	-0.02	0.07
-0.48	-1.11	0.18	0.15	0.16
-0.55	-1.22	0.13	0.11	0.17
-0.67	-1.96	0.5	0.62	0.13
0.9	-1.24	0.66	3.04	0.52
1.15	-0.59	0.29	2.9	0.16

-1	-3.36	0.65	1.36	0.7
-1.93	-5.69	0.51	1.83	0.08
-0.36	-0.99	0.4	0.26	0.14
-0.44	-2.41	0.93	1.53	0.64
0.62	-1.89	0.9	3.14	0.9
-0.56	-3.57	0.96	2.45	0.03
0.99	-1.14	0.58	3.12	0.58
